# Geographic distribution, genetic diversity, and virulence determinants of Shiga toxin-producing *Escherichia coli*: a descriptive review of the retrieved literature

**DOI:** 10.1007/s42770-026-02129-x

**Published:** 2026-11-11

**Authors:** Yan Lucas Matias Viana, Pedro Panzenhagen, Carlos Adam Conte Junior, Hilana Ceotto Vigoder, Gustavo Luis de Paiva Anciens Ramos

**Affiliations:** 1 Specialization in Food Safety and Nutritional Quality Management, Federal Institute of Education, Science and Technology of Rio de Janeiro, Rio de Janeiro, RJ Brazil; 2 Graduate Program in Veterinary Hygiene, Faculty of Veterinary Medicine, Fluminense Federal University (UFF), Vital Brazil Filho, Niterói, RJ Brazil; 3 Institute of Chemistry (IQ), Federal University of Rio de Janeiro (UFRJ), Rio de Janeiro, RJ 21941-909 Brazil; 4 Federal Institute of Education, Science and Technology of Rio de Janeiro, Rio de Janeiro, RJ Brazil; 5 Department of Food Science, Federal Fluminense University (UFF), Niterói, RJ Brazil

**Keywords:** STEC, Molecular epidemiology, Serotypes, Antimicrobial resistance, One health

## Abstract

Shiga toxin-producing *Escherichia coli* (STEC) is an important foodborne pathogen associated with a wide range of clinical manifestations, from mild gastroenteritis to severe disease. This study aimed to synthesize the evidence retrieved on the reported occurrence and geographic distribution of STEC across different matrices, as well as their genetic diversity, virulence determinants, clinical manifestations, and antimicrobial resistance profiles. A descriptive, non-exhaustive review was conducted based on studies published between 2010 and 2025, resulting in the inclusion of 55 articles after application of the eligibility criteria. The included studies originated from different geographic regions, although the evidence was markedly concentrated in the Americas, particularly Brazil. STEC was investigated across human, animal, food, and environmental sources. Within the retrieved literature, *stx2* and *stx1* were widely reported, and *stx2a*, *stx2c*, and *stx2d* were among the recurrently reported *stx2* subtypes. O157 and non-O157 STEC were both represented across the included studies. These findings describe reporting patterns within the retrieved literature and should not be interpreted as frequencies of occurrence among studies specifically testing each characteristic or as prevalence among STEC isolates. The included studies also reported diverse sequence types and additional virulence determinants, including *eae* and *ehxA*. Clinical information was available in only a subset of studies and included manifestations ranging from diarrhea and bloody diarrhea to hemolytic uremic syndrome (HUS). Antimicrobial resistance was investigated in a subset of the included studies using phenotypic and/or genotypic approaches. Resistance phenotypes and resistance-associated genetic determinants were reported among STEC isolates in different study populations; however, heterogeneity in analytical populations, antimicrobial panels, testing methods, and reporting approaches precluded overall or pooled estimates of antimicrobial resistance. Overall, the findings highlight the diversity of STEC reported across different sources and geographic settings and reinforce the importance of integrated molecular and epidemiological approaches within a One Health framework.

## Introduction

Shiga toxin-producing *Escherichia coli* (STEC) is one of the major foodborne pathogens of global relevance, widely associated with human infections ranging from mild gastroenteritis to severe manifestations such as hemorrhagic colitis and hemolytic uremic syndrome (HUS). Recent surveillance data illustrate this public health burden: in 2023, 10,901 confirmed STEC infections were reported across 30 European Union/European Economic Area (EU/EEA) countries, corresponding to a notification rate of 3.2 cases per 100,000 population, the highest recorded since the beginning of EU/EEA-wide surveillance. In the same year, 522 confirmed HUS cases were reported, with most occurring among children and adolescents. The pathogenicity of these bacteria is primarily associated with the production of Shiga toxins, encoded by the *stx1* and *stx2* genes, in addition to other virulence factors involved in intestinal adhesion and colonization, including *eae*, which encodes the adhesin intimin, and *ehxA*, which encodes enterohemolysin [28, 31]. Shiga toxins contribute to STEC pathogenicity by binding to specific glycolipid receptors on susceptible host cells and inhibiting protein synthesis, leading to cellular injury. Systemic toxin-mediated endothelial damage, particularly in the renal microvasculature, is an important mechanism underlying severe complications such as HUS [42]. Different Shiga toxin subtypes have been described among STEC strains and may contribute, together with other bacterial and host factors, to differences in pathogenic potential and clinical outcomes [6, 33].

The dissemination of STEC occurs across multiple matrices, including animals, food, and the environment, reflecting its zoonotic nature and public health relevance. Ruminants, particularly cattle, are recognized as important reservoirs of these bacteria, often exhibiting asymptomatic colonization, with the terminal rectum and rectoanal junction representing important colonization sites in cattle. Colonized animals may shed STEC through feces, contributing to contamination of the farm environment, water, and food and facilitating pathogen dissemination throughout the food production chain [22, 24]. The presence of STEC in products of animal origin, such as meat and milk, as well as in production-related environments including water, feces, and transport surfaces, highlights the complexity of transmission routes and the risk of human exposure [12, 17, 40]. Foodborne exposure is not restricted to products of animal origin, as fresh produce and leafy vegetables may also serve as vehicles for STEC transmission when contaminated through environmental or production-related routes [64].

From an epidemiological perspective, the diversity of serotypes and genetic profiles associated with STEC is noteworthy, including both classical strains such as O157:H7 and a broad range of non-O157 serotypes reported in different geographic settings, including O26, O45, O103, O111, O121, and O145, which are among the major non-O157 serogroups associated with human infections [7, 26, 29]. Beyond serotype classification, sequence typing and genomic approaches have demonstrated substantial genetic diversity among STEC strains from human, animal, food, and environmental sources. Multiple sequence types have been identified among STEC isolates, illustrating the heterogeneity of their genetic backgrounds and supporting the use of molecular and genomic approaches to characterize strains beyond traditional serotyping.

Despite advances in the molecular and epidemiological characterization of STEC, important gaps remain in the integrated understanding of the geographic distribution of the available evidence, genetic diversity, and characteristics of STEC reported across different matrices. The heterogeneity of studies, combined with the diversity of detection and typing methods, limits direct comparisons among studies and complicates the synthesis of findings across different sources and geographic settings.

Therefore, this review aimed to synthesize the evidence retrieved through the predefined search strategy from studies published between 2010 and 2025 regarding the reported occurrence and geographic distribution of STEC across food, animal, environmental, and human sources, and to describe the genetic diversity, major virulence determinants, clinical manifestations, and antimicrobial resistance profiles reported in the included studies.

## Methodology

### Search strategy

Shiga toxin-producing *Escherichia coli* (STEC) was investigated through a descriptive, non-exhaustive review. The identification, screening, eligibility assessment, and inclusion of records were documented using the PRISMA 2020 framework to provide transparency regarding the study-selection process [48]. An electronic search was performed in PubMed, SciELO, Embase, Scopus, and Web of Science on November 18, 2025, considering studies published between January 2010 and the search date.

The search strategy was based on combinations of free-text terms related to Escherichia coli, Shiga toxin-producing Escherichia coli (STEC), Shiga toxin, and sequence typing. The principal search concepts included “Escherichia coli”, “E. coli”, “STEC”, “Shiga toxin”, “ST11”, and “sequence type 11”. Because the original database-specific search histories, search fields, and filters were not retained in sufficient detail to allow complete retrospective reconstruction of the searches as executed, the search expression reported here should be considered a representation of the principal search concepts rather than an exact reproducible database-specific search string.

The original screening records indicated that 194 records were retrieved across the five databases. However, the database-specific counts reported in the PRISMA flow diagram could not be independently reconstructed retrospectively from the original database search histories. Therefore, these counts are presented as the records documented during the original study-selection process rather than as results generated by re-execution of the simplified search expression described above.

Although ST11 was included among the search terms, it was not used as an eligibility criterion and was not required for study inclusion. Sequence type information, including ST11 when available, was extracted only as part of the genetic characterization of STEC strains reported in eligible studies. Because the explicit inclusion of ST11 in the original search strategy may have preferentially increased the retrieval of studies reporting this sequence type, its frequency within the retrieved literature was not interpreted comparatively as evidence of predominance over other sequence types.

The review question and eligibility framework were structured according to the Population–Concept–Context (PCC) framework. The Population comprised studies reporting STEC or STEC isolates/strains. The Concept encompassed the occurrence and geographic distribution reported in the literature and the microbiological and genetic characterization of STEC, including serotypes/serogroups, virulence determinants, sequence types, molecular typing methods, clinical information, and antimicrobial resistance when available. The Context included human, animal, food, and environmental matrices across different geographic settings.

The screening of titles and abstracts was conducted independently by two reviewers, and disagreements were resolved through discussion with a third reviewer.

Because the original database searches were not rerun with additional terminology, terms such as VTEC, EHEC, shigatoxigenic, additional serogroups, reservoirs, foodborne outbreaks, and specific environmental sources were not systematically incorporated into all database queries. This is acknowledged as a limitation of the retrieval strategy. Accordingly, this study is framed as a descriptive, non-exhaustive review, and its findings represent only the body of literature retrieved through the original search process. They should not be interpreted as a comprehensive representation of all STEC studies published during the period considered.

### Focus questions

This review was designed to address a set of key questions related to the occurrence and geographic distribution of Shiga toxin-producing *Escherichia coli* (STEC) reported in the retrieved literature, as well as its main microbiological, genetic, clinical, and epidemiological characteristics and its relevance to public health. The guiding questions of this review were: (I) How are the studies reporting STEC distributed across different countries, geographic settings, and human, animal, food, and environmental sources? (II) Which genetic characteristics, serotypes/serogroups, sequence types, Shiga toxin subtypes, and other virulence determinants have been reported among STEC strains in the included studies? (III) Which laboratory methodologies have been used to detect and characterize STEC strains, including molecular and genomic approaches such as PCR, MLST, whole-genome sequencing (WGS), and serotyping? (IV) Which clinical manifestations have been reported in studies providing clinical information, and what limitations exist regarding the interpretation of relationships between virulence profiles and clinical outcomes? (V) Which phenotypic and genotypic antimicrobial resistance characteristics have been reported in studies that specifically assessed antimicrobial resistance? (VI) What are the main methodological and evidence gaps identified in the included literature, particularly regarding geographic representation, molecular characterization, clinical information, and antimicrobial resistance?

When reported in the included studies, sequence type (ST), including ST11, was recorded during the data extraction stage, allowing for descriptive analysis of the genetic diversity of STEC strains. ST11 was not treated as a specific review question or eligibility criterion**.**

Together, these questions guided the selection, analysis, and synthesis of the studies included in this review, without assuming that the retrieved literature represents the global prevalence, burden, or complete geographic distribution of STEC.

### Selection criteria

#### Eligibility criteria

Original articles published in English between January 2010 and the search date in 2025 reporting data on the occurrence, detection, isolation, or microbiological and molecular characterization of Shiga toxin-producing *Escherichia coli* (STEC) were considered eligible. Epidemiological and observational studies involving human, animal, food, or environmental matrices were included in the descriptive synthesis of STEC occurrence, geographic distribution, sources of isolates, and epidemiological characteristics.

Experimental, mechanistic, methodological, genomic, surveillance, or outbreak-related studies investigating STEC strains were considered separately when they provided complementary information on molecular, genomic, epidemiological, clinical, or functional characteristics relevant to virulence, toxin regulation, bacteriophages, or other genetic determinants. Experimental, mechanistic, and methodological studies without an epidemiological sampling framework were not included in analyses of occurrence, geographic distribution, source attribution, or prevalence.

When available, information on serotype/serogroup, sequence type (ST), molecular/genomic methods, virulence determinants, plasmids and other mobile genetic elements, pathogenicity islands, clinical manifestations, and antimicrobial resistance was extracted for descriptive analysis.

#### Exclusion criteria

Studies were excluded if they did not investigate *Escherichia coli* in a context relevant to STEC or did not present original data related to the detection, isolation, or characterization of STEC or STEC-associated serotypes. Studies in which the STEC pathotype could not be confirmed were retained only when their findings provided relevant contextual information for the epidemiological or microbiological characterization of STEC and were not considered in STEC-specific analyses. Studies published in languages other than English were also excluded, as were review articles, case reports, editorials, letters to the editor, and conference abstracts that did not provide sufficient primary data for analysis.

Additionally, duplicate records identified across databases were excluded, as were studies for which the full text was unavailable for analysis.

### Data extraction and synthesis

Data were extracted independently by two reviewers using a standardized form. The following information was collected: first author, year of publication, country, study category, source of isolates, serotype/serogroup, sequence type (ST), molecular/genomic methods, virulence determinants, plasmids/mobile genetic elements, pathogenicity islands, clinical manifestations, antimicrobial resistance, and main outcomes. Disagreements were resolved by discussion and consensus.

The included studies were summarized descriptively according to their main characteristics, geographic distribution, study categories, sources of isolates, laboratory methods, serotypes/serogroups, sequence types, virulence profiles, clinical manifestations, plasmids/mobile genetic elements, pathogenicity islands, and antimicrobial resistance findings. Because of the substantial heterogeneity among studies, no quantitative meta-analysis was performed.

For the descriptive synthesis, the study was considered the primary unit of analysis when summarizing the frequency with which specific characteristics, genes, serotypes, molecular methods, clinical manifestations, or antimicrobial resistance assessments were reported. Therefore, percentages calculated using the 55 included studies as the denominator represent study-level reporting frequencies and should not be interpreted as prevalence among isolates, samples, hosts, or food products. Because the included studies differed substantially in their objectives, analytical targets, and molecular or phenotypic methods, the absence of a variable from an individual study was not assumed to indicate that the variable had been evaluated and yielded a negative result. When the number of studies that specifically evaluated a given variable could be reliably determined from the original publications, findings were interpreted within that analytical subset. When such a denominator could not be established consistently, study-level counts or percentages were interpreted exclusively as patterns of reporting within the retrieved literature rather than as frequencies of occurrence among studies evaluating that variable. Accordingly, “not reported” was distinguished from “not tested” and “not detected” whenever this information could be established from the original study.

Sample-, isolate-, patient-, or animal-level proportions were reported only when explicitly provided by the original studies, with the corresponding numerator and denominator whenever available, and were interpreted exclusively within the respective study population. No pooled prevalence estimates across studies, matrices, countries, or host species were calculated because of substantial heterogeneity in study designs, sampling strategies, sample sizes, and laboratory methods. Experimental, mechanistic, and methodological studies without an epidemiological sampling framework were analyzed according to their specific study objectives and analytical units and were not used to estimate STEC occurrence or prevalence.

### Methodological limitations of the included studies

Given the heterogeneity of the studies included in this review, no formal risk-of-bias scoring system was applied. Instead, methodological limitations relevant to the interpretation of the evidence were considered descriptively. For each included study, the assessment considered the adequacy and completeness of the reported sample size and sampling strategy, the description of laboratory methods used for STEC detection and characterization, and the consistency and completeness of information regarding the analyzed samples or isolates. These elements were examined qualitatively to identify limitations that could affect the interpretation and comparability of individual studies. No numerical score or categorical risk-of-bias classification (e.g., low, moderate, or high risk) was assigned.

These limitations included differences in study design, sample size, sampling strategy, source and type of specimens, laboratory methods used for STEC detection and characterization, and completeness of reporting. Additional sources of heterogeneity included variation in molecular typing approaches, virulence gene panels, antimicrobial susceptibility testing methods, and the amount of epidemiological or clinical information provided by individual studies. The inclusion of different study categories, including observational, experimental, mechanistic, methodological, genomic, surveillance, and outbreak-related investigations, further limited direct comparisons across studies because these designs differed in their objectives, sampling frameworks, and analytical units.

The restriction to studies published in English, the predefined search strategy, and the uneven geographic distribution of the included literature were also considered important limitations of the evidence base. Therefore, the findings of this review should be interpreted as a descriptive, non-exhaustive synthesis of the studies identified by the predefined search strategy, rather than as a comprehensive representation of the STEC literature, a formally weighted assessment of study quality, or a pooled estimate of STEC occurrence, prevalence, or risk. These limitations were considered throughout the interpretation of the findings, particularly when comparing geographic distribution, clinical manifestations, and antimicrobial resistance across studies.

## Results

### Literature search

**Fig. 1:**
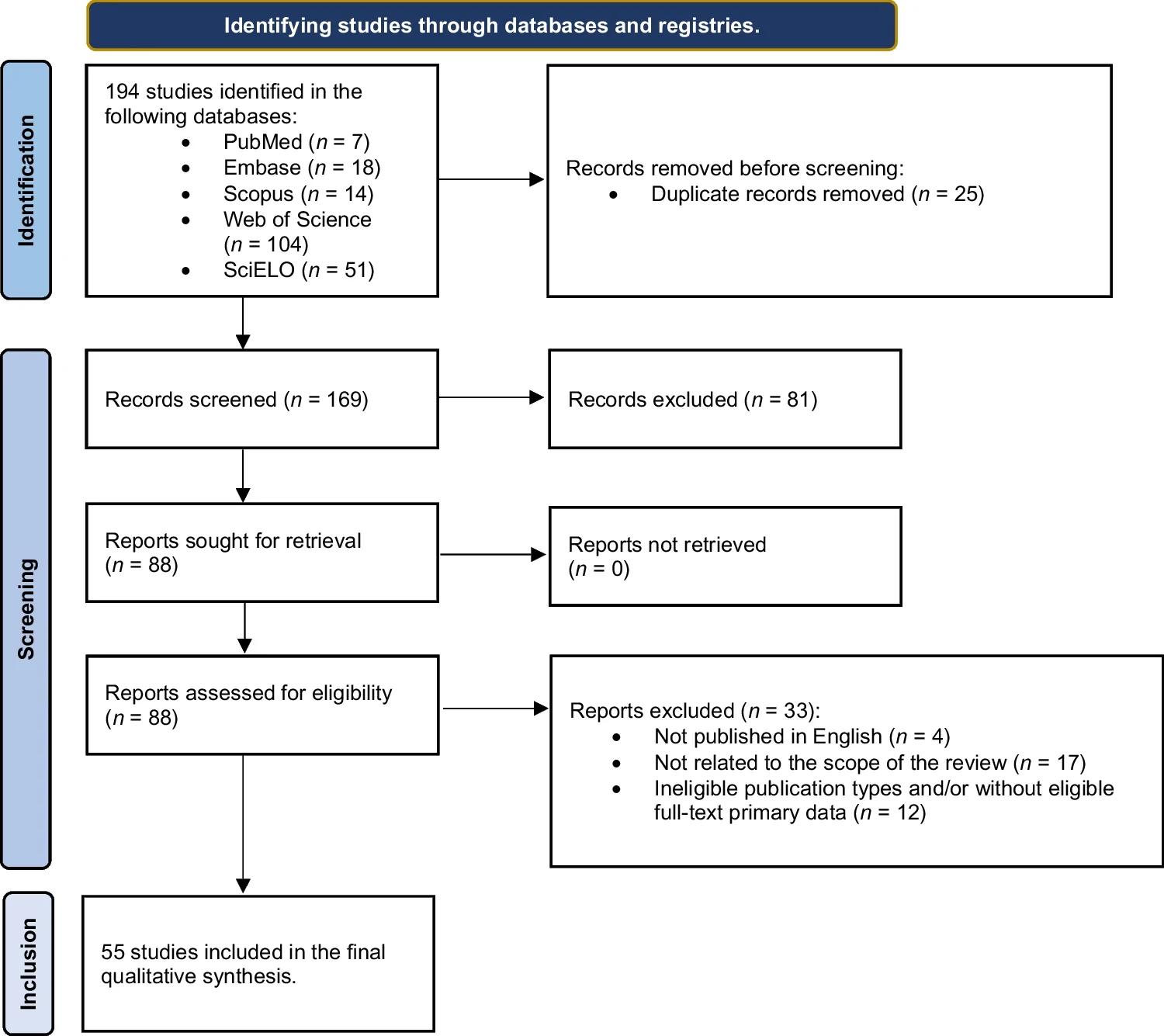
PRISMA 2020 flow diagram of the study selection process. The diagram presents the records documented during the original literature search across PubMed, Embase, Scopus, SciELO, and Web of Science, followed by duplicate removal, title and abstract screening, full-text eligibility assessment, and inclusion of 55 studies in the final descriptive synthesis. Database-specific search histories, fields, and filters from the original search were not retained in sufficient detail to allow complete retrospective reconstruction of the reported database-specific retrieval counts

The identification, screening, eligibility assessment, and inclusion of studies were documented using the PRISMA 2020 framework to provide transparency regarding the study-selection process. The study selection process is presented in the PRISMA flow diagram (Fig. 1), and the characteristics and main findings of the included studies are summarized in Table 1.

**Table 1:** Characteristics and main findings of the studies included in the descriptive review

Country	Year	Study category	Source of isolates	Serotype/serogroup	ST	Molecular/genomic methods	Virulence determinants	Plasmids/mobile genetic elements	Pathogenicity islands	Clinical manifestations	Antimicrobial Resistance	Author
Brazil	2025	Experimental/in vitro	Bovine-derived STEC strains evaluated in vitro	O157:H7	NR	NA	NR	NR	NR	NA	NR	[57]
China	2024	Epidemiological/observational – molecular characterization	Feces from diarrheic lambs	NR	NR	PCR; Kirby–Bauer disk diffusion	*mdh, hlyF, iss, ompA, fimC, iucD, st, lt, stx1, stx2*	NR	NR	Diarrhea	**AMR data reported for the total *E. coli* population (n = 178), not specifically for STEC isolates:** phenotypic resistance to lincomycin, 172/178 (96.63%); tetracycline, 158/178 (88.76%); co-trimoxazole, 143/178 (80.34%); MDR, 167/178 (93.82%). Resistance genes in the total *E. coli* population: *tetA/tetB*, 165/178 (92.70%); *qnr*, 135/178 (75.84%); *sul1*, 112/178 (62.92%); *blaTEM* and *blaCTX-M*, ND. STEC-specific phenotypic AMR proportions were not separately reported	[21]
Brazil	2021	Epidemiological/observational – molecular characterization	Cattle and sheep feces	NR	NR	PCR	**Bovine isolates*:**** stx1a, stx1c, stx2a, stx2b, stx2c, stx2d;* **ovine isolates:** *stx1a, stx1c, stx2a, stx2b, stx2c, stx2g*	NR	NR	NA	Resistance to ≥ 1 antimicrobial: 57/59 bovine isolates (96.6%) and 42/47 ovine isolates (89.4%); MDR: 40/59 bovine isolates (67.8%) and 28/47 ovine isolates (59.6%)	[1]
Brazil	2020	Epidemiological/observational – detection study	Frozen chicken carcasses	NR	NR	Multiplex PCR	*stx1:* ND*; stx2:* ND*; eae:* detected in aEPEC isolates	NT	NT	NA	NA for STEC; antimicrobial susceptibility evaluated in aEPEC isolates	[15]
Brazil	2019	Epidemiological/observational – molecular characterization	Feces from buffalo calves with and without diarrhea	NR	NR	PCR	*EAST-1, enterohemolysin, Saa, CNF2, F41, F5, STa, intimin, Stx1, Stx2*	NR	NR	Diarrheic and non-diarrheic calves	NR	[19]
Brazil	2019	Epidemiological/observational – detection study	Raw milk, water, and cattle feces	NR	NR	Multiplex PCR	*stx1, stx2, eae* (genes investigated for diarrheagenic *E. coli* pathotyping)	NR	NR	NA	NR	[53]
Brazil	2018	Epidemiological/observational – molecular characterization	Cattle feces	NR	NR	Triplex PCR; phylogenetic group classification	*stx1* and *stx2* detected; distribution evaluated according to season, animal age, and production scale	NR	NR	NA	NR	[22]
Brazil	2018	Epidemiological/observational – molecular characterization	Feces from dogs with and without diarrhea	NR	NR	PCR; phylogenetic group classification	STEC isolates: *stx2* (1/37 virulence-positive *E. coli* isolates) and *stx2e* (1/37); additional virulence genes were evaluated in non-STEC *E. coli* isolates	NR	NR	Diarrheic and non-diarrheic dogs	NR	[18]
Brazil	2017	Epidemiological/observational – detection and molecular characterization	Artisanal mozzarella cheese	non-O157	NR	PCR; O157 latex agglutination	*stx1*: 14/147 *E. coli* isolates (9.5%); *eae*: 11/14 STEC isolates (78.6%); *stx2*: ND	NR	NR	NA	NR	[12]
Brazil	2015	Epidemiological/observational – molecular characterization	Rectal swabs from healthy sheep	O157:H7 and non-O157	NR	PCR; antimicrobial susceptibility testing by disk diffusion	*stx1*: 47/90 STEC isolates (52.2%); *stx2*: 30/90 (33.3%); *stx1* + *stx2*: 13/90 (14.5%); *saa*: 43/90 (47.7%); *cnf1*: 29/90 (32.2%); *eae* and *ehxA* also investigated	NR	NR	Asymptomatic/healthy sheep	Resistance to ≥ 1 antimicrobial: 75/90 STEC isolates (83.3%); resistance to two antimicrobials: 11/90 (12.2%); resistance to ≥ 3 antimicrobials: 6/90 (6.7%)	[23]
Brazil	2015	Epidemiological/observational – molecular characterization	Dairy cow feces	O5, O6, O8, O9, O20, O26, O55, O75, O86, O91, O101, O146, O149, O153, O157, O158, O172	NR	PCR; antimicrobial susceptibility testing	*stx1*: 90/561 *E. coli* isolates (16.0%); *stx2*: 97/561 (17.3%); *stx1* + *stx2*: 37/561 (6.6%); *eae*	NR	NR	NA	*stx1*-positive isolates: streptomycin, 77/90 (85.6%); kanamycin, 65/90 (72.2%); nalidixic acid, 63/90 (70.0%). *stx2*-positive isolates: streptomycin, 71/97 (73.2%); kanamycin and nalidixic acid, 68/97 each (70.1%)	[2]
Brazil	2015	Experimental/in vitro – survival study	STEC O157:H7 strain RJ581 and reference strain EDL933; experimentally inoculated into Minas Frescal cheese	O157:H7	NR	NR	NR	NR	NR	NA	NR	[25]
Brazil	2014	Experimental – composting survival study	STEC isolates from naturally colonized sheep; ovine manure used in the experimental study	non-O157	NR	PCR; O157:H7 latex agglutination	*stx1, stx2, eae*	NR	NR	Asymptomatic/healthy sheep	STEC *stx2*-positive isolates (n = 114) showed resistance to multiple antimicrobials; susceptibility was evaluated against 10 antimicrobial agents by disk diffusion	[39]
Brazil	2014	Epidemiological/observational – molecular screening	Bovine raw milk	NR	NR	Multiplex PCR	*stx1*: 1.9% of *E. coli*-positive milk samples; *stx2*: 28.3%; STEC markers (*stx1* and/or *stx2*): 31.1%	NR	NR	NA	NR	[63]
Brazil	2014	Epidemiological/observational – molecular screening	Feces from diarrheic and non-diarrheic cats; urine from cats with UTI	NR	NR	PCR	*stx1: ND; stx2:* ND*; eae* detected in 3 *E. coli* isolates (all *sfa* + *eae*); afa: ND; *pap* and *sfa* also evaluated	NR	NR	Diarrhea; urinary tract infection (UTI)	NR	[11]
Brazil	2014	Epidemiological/observational – molecular characterization	Rectal swabs from healthy dairy cattle	O157:H7	NR	PCR; serotyping; phylogenetic group classification	O157:H7 isolates:* stx2, eae, ehxA, saa, cnf1*	NR	NR	Asymptomatic/healthy cattle	NR	[24]
Brazil	2014	Epidemiological/observational – detection study	Minas Frescal cheese	O157:H7	NR	PCR; ELISA (VIDAS ECO O157®); serological confirmation	**O157:H7** detected; *stx1/stx2* not investigated/demonstrated; not considered STEC-specific	NR	NR	NA	NR	[13]
Brazil	2014	Epidemiological/observational – molecular screening	Minas Frescal cheese	NR	NR	PCR	*stx1:* ND*; stx2:* ND*; eae:* ND*; pap:* ND*; afa:* ND*; sfa:* 1/330 *E. coli* isolates (0.3%)	NR	NR	NA	MDR among *E. coli* isolates: 16.6% from SIF-inspected cheese; 8.0% from non-SIF cheese; 30.0% from non-SIF cheese with added spices. Predominant resistance: cephalothin, tetracycline, and streptomycin	[46]
Argentina	2013	Epidemiological/observational – molecular characterization	Rectal swabs and carcass samples from healthy cattle	STEC: O172:H−, O91:H21, O178:H19, O2:H5, and non-typeable isolates; O150:H8 identified among non-STEC pathogenic *E. coli*	NR	PCR; RAPD; O:H serotyping	Among 12 pathogenic *E. coli* isolates: *stx1*, 3/12; *stx2*, 3/12; *eae*, 2/12; *astA*, 4/12. *eae* was ND among STEC isolates	NR	NR	Asymptomatic/healthy cattle	Low overall resistance among the 12 pathogenic E. coli isolates; all were susceptible to ciprofloxacin and sulfamethoxazole–trimethoprim, whereas most were resistant to colistin. STEC-specific resistance data were not separately reported	[51]
Brazil	2013	Epidemiological/observational – molecular characterization	Stable flies *(Stomoxys calcitrans)*: body surface, mouthparts, and abdominal digestive tract	NR	NR	Multiplex PCR	*stx1*: 6/6 STEC isolates; *stx2*: 5/6; *eae*: 1/6	NR	NR	NA	NR	[14]
United States	2023	Experimental/mechanistic – mouse model and phage regulation study	Human clinical STEC O157:H7 isolates and derived experimental strains; mouse model used for virulence assays	O157:H7	NR	PCR; whole-genome sequence analysis; targeted mutagenesis	*stx2a*; *stx2c*	*stx2a*-encoding bacteriophages	NR	NA – experimental mouse model	NA	[4]
United States	2023	Experimental/mechanistic – mouse virulence and *Stx* phage study	Human clinical STEC O157:H7 isolates and derived experimental strains; mouse model used for virulence assays	O157:H7	NR	PCR; RT-qPCR; comparative genomic analysis; targeted mutagenesis	*stx2a*; *stx2c*	*stx2a*-encoding bacteriophages	NR	NA – experimental mouse model	NA	[3]
United States	2023	Genomic/molecular characterization of selected strains carrying novel/rare *stx2* subtypes	Human clinical isolates	O158, O162, O32, ONT, O33, O183, O23, O1	ST5662, ST5350, ST5736, ST491, ST5923, ST657, ST70, ST1385	WGS; in silico MLST; PCR; *stx* subtyping; prophage analysis	stx2j, stx2m, stx2n, stx2o	*stx2 -*associated prophages	NR	NR	NR	[33]
England	2023	Epidemiological/observational – genomic surveillance and clinical characterization	Human fecal specimens	non-O157	ST583, ST122, ST6044, ST7703, ST35, ST526, ST713, ST722, ST1877, ST10, ST20, ST327, ST95, ST589, ST2678, ST12706, ST9955	WGS; in silico MLST; phylogenetic analysis	*stx2f, eae, astA, bfpA, cdt*	*stx2f*-encoding prophage; 85-kbp IncFIB plasmid	NR	Diarrhea, bloody diarrhea, vomiting, fever, abdominal pain; STEC-HUS: 7/40 cases with available clinical data (17.5%)	NR	[20]
Norway	2016	Molecular/genomic characterization – clinical isolates	Human clinical isolates	O157:H7 and non-O157	NR	Multiplex PCR; genomic analysis	*stx2a*: 20/20 STEC isolates; *eae*: 15/20; *ehxA*: 15/20; *aggR*: 3/20; *aaiC*: 3/20	*stx2a*-encoding bacteriophages	LEE (present/absent according to strain)	NR	NR	[47]
Germany	2025	Experimental/mechanistic – *Stx2a* expression and toxin-subunit production	Human clinical and food-derived STEC strains used experimentally	O157:H7, O157:H−, O103:H−, O26:H11, O104:H4, O113:H21	NR	qPCR; quantitative ELISA; native PAGE; Western blot	*stx2a* (*stxA2a, stxB2a*)	NR	NR	HUS-associated clinical isolates; NA for foodborne isolate	NR	[45]
Germany	2025	Genomic/molecular characterization – food and animal isolates	Roe deer meat and cattle feces	O128:H2; O150:H2	ST306; ST25	WGS (NGS); PCR*; *in silico MLST; genomic analysis	**O128:H2/ST306:** *stx1a, stx1c, eae, nleB, ehxA;* **O150:H2/ST25:** *stx1a, stx2a, ehxA*	*stx1*-associated prophages; insertion sequence associated with disruption of *stxA1a*	NR	NA	NR	[52]
Sweden and Denmark	2021	Genomic/molecular characterization – clinical isolates	Human clinical isolates from diarrheal patients and an asymptomatic carrier	O148:H39; O96:H19	ST5825;ST99	WGS; in silico MLST; *stx* subtyping; phylogenetic analysis	*stx2m, iha, astA, terC, hra, chuA, eilA, traT, kpsMII_K5, mchB, mchC, mchF, celB, iss, lpfA, ompT*	NR	NR	Diarrhea (2/3 cases); asymptomatic carriage (1/3)	Genotypic: *mdf(A)* detected in 3/3 isolates. Phenotypic: all three isolates were susceptible to all antimicrobials tested, including azithromycin	[7]
United States	2012	Comparative genomic/mechanistic study of selected LEE-negative STEC strains	Nine LEE-negative STEC isolates from human and animal sources; four LEE-positive STEC genomes used as references	O2:H25; O48:H21; O91:H21; O73:H18; O174:H8; O118:H12; O8:H19; O174:H21; O139:H1	NR	Whole-genome sequencing; comparative genomic analysis; PCR	*stx2g, stx1a, stx2a, stx2d1, stx2d2, stx2d, stx1c, stx2b, stx1d, stx2c, stx2e, ehxA, saa*	*stx*-encoding bacteriophages; pO113-like plasmid	LEE: absent in the nine LEE-negative STEC isolates	HUS; bloody diarrhea; abdominal cramps; SIDS; edema disease; healthy/asymptomatic cattle	NR	[59]
Brazil	2010	Phenotypic/molecular characterization – previously isolated STEC strains	Healthy cattle feces (37/38 strains) and a child with diarrhea (1/38)	Multiple non-O157 serotypes	NR	Plasmid profiling; PCR-based characterization from previous studies	STEC status previously confirmed by *stx* PCR/cytotoxicity; enterohemolysin production: 11/38 strains (29%)	One or more plasmids detected in 95% of the 38 STEC strains; detailed plasmid profiling was reported for 36 strains, including 18/36 (50%) with a single ~ 90-kb plasmid	NR	Diarrhea in human source cases; healthy/asymptomatic cattle	NR	[58]
China	2018	Epidemiological/observational – molecular and genomic characterization	Intestinal contents of healthy wild Himalayan marmots (Marmota himalayana); 6 STEC recovered from 200 samples (3%)	O102:H18 (4/6); Orough:H8 (1/6); O168:H14 (1/6)	ST3693 (4/6); ST26 (1/6); ST718 (1/6)	PCR; *stx2* subtyping; WGS; MLST; phylogenomic analysis	**O102:H18/ST3693:** *stx2h*, *paa* (4/6); **Orough:H8/ST26:** *stx2a*, *ehxA*, *saa* (1/6); **O168:H14/ST718:** *stx2g*, *astA*, *saa* (1/6)	*stx2h*-encoding prophage	LEE absent (*eae*-negative)	Asymptomatic/healthy wild marmots	NR	[6]
Brazil	2023	Epidemiological/observational – molecular characterization and antimicrobial susceptibility	Conventional broiler chickens: cloacal samples (16 STEC) and carcasses (19 STEC); STEC not detected in organic broilers	NR	NR	PCR	*eae, stx1 and stx2* investigated by PCR for EPEC/STEC pathotyping	NR	NR	NA	MDR detected in 1/35 STEC isolates (2.86%). Among 21 MDR E. coli isolates, one belonged to the STEC pathotype. Antimicrobial susceptibility results for individual drugs were reported primarily for the overall *E. coli* population	[34]
Brazil	2020	Epidemiological/observational – molecular characterization	Cloacal samples and broiler carcasses	NR	NR	PCR	Among 35 STEC isolates: *stx1* alone, 2/35 (5.6%); *stx2* alone, 2/35 (5.6%); *stx1* + *stx2*, 7/35 (20%); *stx1* + *eae*, 23/35 (66%); *stx2* + *eae*, 1/35 (2.8%); *stx1* + *stx2* + *eae*: ND	NR	NR	NA	*mcr-1*: 2/35 STEC isolates (5.7%)	[35]
Brazil	2019	Experimental/in vitro – antimicrobial activity of enterocin against STEC	STEC strain from a bacterial culture collection	NR	NR	PCR	*stx1, stx2*	NR	NR	NA	NA	[60]
Brazil	2018	Experimental – anaerobic codigestion of dairy cattle manure	Dairy cattle manure subjected to experimental anaerobic codigestion	NR	NR	PCR	STEC monitored by PCR during anaerobic codigestion; *stx2* and *eae* detected during the experiment	NR	NR	NA	NA	[9]
Iran	2015	Epidemiological/observational – prevalence and molecular characterization	Wild captive and companion birds; 657 E. coli isolates from 219 birds representing 38 species	NR	NR	Multiplex PCR; *stx2f*-specific PCR	STEC detected in 4/219 birds (1.8%); all STEC isolates carried *stx2f* and *eae*. Conventional *stx1/stx2* PCR: ND in these isolates; *stx2f* detected by subtype-specific PCR	NR	NR	NR	NT	[32]
Japan	2013	Molecular characterization – virulence profiling and population genetic analysis	Human clinical isolates, cattle feces, and food isolates	O157 and multiple non-O157 serogroups	NR	PCR; population genetic analysis	Virulence genes were evaluated by PCR; detected determinants varied among isolates* (stx1, stx2a, stx2c, stx2d, stx2e, stx2f, eae, tir, espB, espD, iha, saa, subA, ehxA, espP, katP, stcE)*	NR	LEE-associated genes (*eae, tir, espB, espD*) evaluated	NR	NT	[31]
Spain	2012	Molecular characterization – environmental STEC isolates	Fecally polluted water environments (cattle wastewater and sewage)	O157:H7 and multiple non-O157 serotypes	NR	PCR; sequencing of PCR products	*stx1, stx2* variants, *cdt, hlyA, saa, eae*; LEE-encoded and non-LEE-encoded T3SS effector genes	NR	LEE-associated effector genes evaluated	NA	NT	[40]
Multicenter/international	2012	Methodological – development and multicenter validation of *stx* subtyping	Reference/prototypic STEC strains	Multiple serotypes	NR	Sequence-based analysis; PCR subtyping; multicenter validation	*stx1a, stx1c, stx1d, stx2a, stx2b, stx2c, stx2d, stx2e, stx2f, stx2g*	NR	NA	NA	NA	[54]
Canada	2022	Genomic characterization of selected STEC isolates carrying atypical *stx2* variants	Food and human clinical isolates	O157:H7; O8:H28; O146:H21; O170:H18; O85:H1; O158:H23; O33:H14	NR	Illumina and Oxford Nanopore WGS; PCR; Sanger sequencing; ELISA; Vero cell cytotoxicity assay	*stx1a*; truncated *stx2a*; *stx2a* variant; *stx2b* variant; *stx2h*; *stx2j*; *stx2o*	*stx-*associated prophages	NR	NR	NA	[27]
Italy and France	2016	Methodological – development and validation of HReVAP for STEC characterization	Human clinical and food isolates	O157, O26, O111, O103, O145, O121, and multiple other serogroups	NR	HReVAP: real-time PCR followed by melting-curve analysis of 91 virulence-associated genes	91 virulence-associated genes investigated; presence and allelic profiles varied among strains	NR	LEE (38 targets), OI-122 (12 targets), and OI-57 (41 targets) investigated	NR	NT	[43]
Brazil	2024	Epidemiological/observational – occurrence and genomic characterization	Ground beef; one STEC isolate recovered from 248 samples (0.4%)	O157:H7	ST11 (STEC O157:H7 isolate recovered from ground beef)	Multiplex PCR; WGS; MLST; Vero cell cytotoxicity assay	*stx1, stx2, eae* and *ehx* detected by PCR; WGS identified *stx1a-*O157*, stx2a-*O157*, eae-g01-gamma* and *ehxA,* among other virulence determinants	IncFIB(AP001918); IncFII	NR	NA	No acquired antimicrobial resistance genes detected; phenotypically susceptible to the antimicrobials tested. Chromosomal mutations were identified in *gyrA*, *gyrB*, *parC*, *parE*, *pmrA*, *pmrB*, and *folP *	[37]
Argentina	2023	Epidemiological/observational – occurrence and genomic characterization	Bovine carcasses; 39 STEC isolates recovered from 20 establishments (800 carcasses sampled); human HUS-associated comparator strain	O157:H7; O130; non-typeable O serogroup	ST11 (O157:H7 bovine carcass-derived isolate and human HUS-associated comparator isolate), plus ST156, ST192, ST297, ST300, ST332, ST441, ST443, ST446, ST677, ST1172, ST1248, ST1679, ST1727, ST2387, ST2520, ST2602, ST3695, ST6190, ST6661, and ST7616	Multiplex PCR; serotyping; WGS; MLST; SNP analysis	**O157:H7:** *stx2, eae, ehxA, katP, espP, stcE,* ECSP_0242/1773/2687/2870/2872/3286/3620; **non-O157:** *stx1*	NR	NR	NA for isolates recovered from carcasses/truck environment; HUS-associated O157 strain included as a comparator	NT	[17]
Uruguay	2023	Epidemiological/observational – occurrence, phenotypic and genomic characterization	Bovine carcasses; 39 STEC isolates from 20 establishments (800 carcasses sampled)	21 O-groups and 13 H-types; O130:H11 (6/39), O174:H28 (5/39), O22:H8 (5/39), and O157:H7 (1/39), among others	Multiple STs; O157:H7 isolate: ST11	Multiplex PCR; WGS*; *in silico serotyping; MLST	*stx2a*, *stx2c*, or *stx2d:* 33/39 isolates (84.6%); *eae*: 2/39; *lpfA*: 38/39; *ompA*: 39/39; *ompT*: 39/39; *iss*: 38/39; *terC*: 39/39; additional virulence determinants varied among isolates	Plasmid-associated virulence genes included *epeA*, *espP* and *ehxA*	NR	NA	Among 39 STEC isolates, no phenotypic resistance was detected by disk diffusion; one isolate showed resistance to tetracycline and minocycline by MicroScan. Resistance genes associated with tetracycline/minocycline, sulfonamides, streptomycin, and fosfomycin were detected	[49]
South America (Chile, Argentina, Ecuador, Paraguay, and Uruguay)	2021	Genomic epidemiology – comparative genomic analysis	STEC genomes from cattle (51/130), beef (48/130), and humans (31/130) from South America	O157:H7 and multiple non-O157 serotypes	ST11, ST16, ST21, ST223, ST443, ST677, ST679, ST2388, and multiple additional STs	WGS*; *in silico MLST and serotyping; virulome and resistome analysis; SNP-based phylogenomic analysis	*stx1a, stx1c, stx2a, stx2b, stx2c, stx2d, stx2g, eae, ehaA, iha, lpfA* and additional virulence genes	NR	LAA-associated genes evaluated	NR	Among STEC isolates, acquired AMR genes were detected at low frequency, including *aph(3″)-Ib*, *aph(6)-Id*, *aph(3′)-Ia*, *tetA/tetB*, *blaTEM-1*, *sul2*, *fosA7*, and *qnrB19*; chromosomal *blaAmpC* was detected in all genomes	[26]
Japan	2019	Epidemiological/observational – genomic epidemiology and surveillance	Human fecal isolates: symptomatic patients (38/65) and asymptomatic food handlers (27/65)	O157:H7 and multiple non-O157 serotypes	ST11, ST21, ST2966, ST1705, ST88, ST2385, ST17, ST675, and additional STs	PCR; WGS; MLST; core-genome SNP analysis	Among 48 isolates belonging to serotypes recovered from patients, *eae, tir, espB, espA, espJ, nleA, nleB, and nleC* were detected in 41–47/48 isolates (85–98%); *saa:* 5/65, all in non-O157 isolates from asymptomatic food handlers. stx subtype profiles varied among isolates	NR	LEE-associated genes evaluated	Bloody diarrhea: 28/38 patients (74%); HUS: 1/38 (2.6%); clinical manifestations unknown for the remaining patients	Acquired AMR genes detected in 18/65 STEC isolates (28%); aminoglycoside resistance genes in 16/65 (25%); sulfonamide/trimethoprim resistance genes in 14/65 (22%); tetracycline resistance genes in 11/65 (17%); blaTEM-1 B in 7/65 (11%); macrolide resistance genes in 3/65 (5%); and phenicol resistance genes in 2/65 (3%)	[5]
United States	2016	Experimental – bacteriophage-mediated horizontal gene transfer	*E. coli* O157:H7 RM8530 and *Enterobacter cloacae* M12X01451 as *Stx*-phage donors; additional *E. coli* and *E. cloacae* strains used as experimental recipients	*E. coli* RM8530: O157:H7; NA for *E. cloacae*	NR	PCR; sequencing; ELISA; Western blot	*stx1a* in *E. coli* O157:H7 RM8530; *stx1e* in *E. cloacae* M12X01451	*Stx1a-* and *Stx1e-*converting temperate bacteriophages	NA	NA	NT	[30]
Norway	2018	Experimental/methodological – investigation of *stx* loss and implications for STEC diagnostics	Human clinical isolates from patients with diarrhea	O26:H11; O103:H2; O103:H?; O145:H28; O145:H25; O157:H −	Multiple STs; ST6130 reported	qPCR; WGS; wgMLST; comparative genomic analysis; culture-based assays	*stx-*positive and *stx*-negative derivatives were investigated; *stx* loss was demonstrated in vivo and in vitro	*stx-*associated prophages; complete or partial loss of the parental prophage was associated with *stx* loss	NR	Infectious diarrhea; bloody diarrhea reported in some patients	NT	[55]
Canada	2018	Experimental/genomic – characterization of stx-converting prophages	STEC strains of human, food, and outbreak-associated origins	O26:H11; O45:H2; O76:H19; O103:H25; O104:H4; O111:NM; O113:H4; O121:H19; O121:NM; O145:NM; O157:H7 and others	NR	PCR; Sanger sequencing; sequence alignment and phylogenetic analysis; reporter plasmid construction; fluorescence microscopy; flow cytometry	*stx* variants characterized in the selected experimental strains	*stx-*converting prophages and reporter constructs used for experimental characterization	NA	NA	NT	[65]
Argentina	2020	Experimental/mechanistic – functional characterization of *nleB*	Cattle, food, and human clinical isolates	O26:H11; O111:H2; O118:H16; O121:H19; O128:NM; O145:NM; O157:H7; O165:NM; O177:NM; O103:H2	NR	PCR; RT-qPCR; *nleB* transcription analysis	*stx1, stx2, eae, nleB, ehxA, saa, subA, katP, espP, stcE, epeA*	Plasmid-associated virulence genes (*ehxA*, *saa*, *subA*, *katP*, *espP*, *stcE*, *epeA*); OI-122-associated *nleB*	Plasmid-associated virulence genes evaluated/detected (OI-122-associated* nleB)*	NA for the experimental analysis; HUS-associated strain used as comparator/reference, where applicable	NT	[10]
United States	2016	Genomic/experimental – comparative characterization of STEC virulence plasmids	Food and human clinical STEC isolates	O36:H14; O136:H16; O168:H8/HNT; O145:H25 and additional STEC serotypes used for comparative plasmid analysis	NR	Plasmid profiling; PCR-based characterization; plasmid DNA extraction and sequencing; comparative plasmid genomic analysis	*stx1a, stx2a, stx2g, ehxA, astA, sta1, tsh, stcE* and additional virulence determinants	pCFSAN004178; pCFSAN004179; pCFSAN004180; pCFSAN004181; additional *ehxA*-encoding virulence plasmids used for comparison	LEE status evaluated; subtype D strains were LEE-negative	HUS associated with the two clinical subtype E isolates	NT	[36]
United States	2019	Surveillance/genomic characterization – food-derived STEC	FDA-regulated foods	Multiple serotypes, including O26:H11, O157:H7, O91:H14, O103:H2, and O111:H −	95 STs, including ST21, ST29, ST11, ST33, ST17, and ST16	WGS; in silico serotyping; MLST; cgMLST; virulome and resistome analyses	*stx1a, stx1c, stx2a, stx2b, stx2c, stx2d, stx2d/e, stx2e, stx2g, eae, ehxA, espP, etpD, toxB, katP, subA, saa, sab*	NR	LEE-associated *eae* evaluated	NA	Acquired antimicrobial resistance genes detected in 33/276 STEC isolates; genotypic resistance profiles determined by WGS	[28]
Finland	2017	Cross-sectional observational – animal reservoir/occurrence and molecular characterization	Feces from slaughtered reindeer; 470 animals sampled	O157:H − and non-O157 STEC	O157:H − : ST11; non-O157 STEC: four additional STs	Real-time PCR; PCR; MLST; phenotypic characterization	**O157:H − STEC:** *stx1a, stx2c, eae, hlyA* (24 isolates); one additional O157:H − ST11 isolate lacked *stx* but carried *eae, hlyA, and sfpA.*** Non-O157 STEC:** *stx1a* (8/21) or *stx2b* (13/21); *subAB2-2/subAB2-3* variants identified among *stx2b-*positive isolates	NR	NR	NA	NT	[66]
Germany	2016	Longitudinal/genomic study of persistent STEC colonization in cattle	Fecal STEC isolates from cattle longitudinally monitored on four farms in northeastern Germany	Multiple serotypes; persistent STEC mainly O26:H11, O156:H25, O165:H25, and O182:H25	Multiple STs; persistent STEC: ST21, ST396, ST1705, ST300, ST688, and ST119; sporadic STEC included ST11 and ST1079	WGS; MLST; genoserotyping; DNA microarray; CHEF-PAGE	*stx1, stx2, eae, ehxA, katP,* and *espP* evaluated; virulence profiles varied among isolates	NR	LEE-associated status evaluated through eae and genomic characterization	NA	NT	[8]
United Kingdom	2013	Outbreak investigation/genomic epidemiology	Human and animal EHEC/STEC isolates associated with a 2009 open-farm outbreak in the United Kingdom	O157:H7	ST11	WGS, phylogenetic SNP analysis, SNP typing, and MLVA	*stx2a, stx2c, eae* and additional virulence-associated genes	pO157	LEE-associated *eae*	HUS in approximately 22% of 93 outbreak cases	NT	[61]

*NR* not reported; *NT* not tested; *ND* not detected; *NA* not applicable. Information presented for virulence determinants, sequence types, plasmids/mobile genetic elements, pathogenicity islands, and antimicrobial resistance reflects the data explicitly reported in each original study. NR should not be interpreted as absence or as evidence that a given variable was tested and yielded a negative result. Because the analytical targets and molecular panels varied among studies, the absence of a determinant from a Table 1 entry does not necessarily indicate that it was investigated and not detected. Study-specific numerators and denominators are presented only when explicitly supported by the original publication

The original study-selection records documented 194 records retrieved across the five databases: PubMed (*n* = 7), Embase (*n* = 18), Scopus (*n* = 14), SciELO (*n* = 51), and Web of Science (*n* = 104). These database-specific counts correspond to the records documented during the original search and screening process. Because the original database-specific search histories, fields, and filters were not retained in sufficient detail to allow complete retrospective reconstruction of the searches as executed, these counts could not be independently reproduced by re-running the core search expression currently described in Sect. "Search Strategy".

After removal of 25 duplicate records, 169 records were screened based on titles and abstracts, of which 81 were excluded. The remaining 88 reports were sought for retrieval and successfully retrieved for full-text assessment (reports not retrieved, *n* = 0). Following application of the eligibility criteria, 33 reports were excluded: four were not published in English, 17 were not related to the scope of the review, and 12 corresponded to ineligible publication types and/or did not provide eligible full-text primary data. Consequently, 55 studies met the eligibility criteria and were included in the final descriptive synthesis (Fig. 1).

The study-selection process and the corresponding numerical flow are presented in Fig. 1.

### Geographic distribution of the included studies

The 55 included studies represented multiple geographic regions, although their distribution was markedly uneven. Brazil contributed the largest number of studies, followed by the United States, while studies from Argentina, Germany, China, Japan, Norway, Canada, and other countries were less frequently represented. The review also included multinational investigations involving samples or isolates originating from more than one country.

Overall, the available evidence was concentrated in the Americas, particularly Brazil. Therefore, the geographic distribution described in this review represents the distribution of the studies retrieved and included through the predefined search strategy and should not be interpreted as the prevalence, incidence, or burden of STEC in individual countries or as a comprehensive representation of global STEC epidemiology.

### Sources of isolates and study categories

**Fig. 2:**
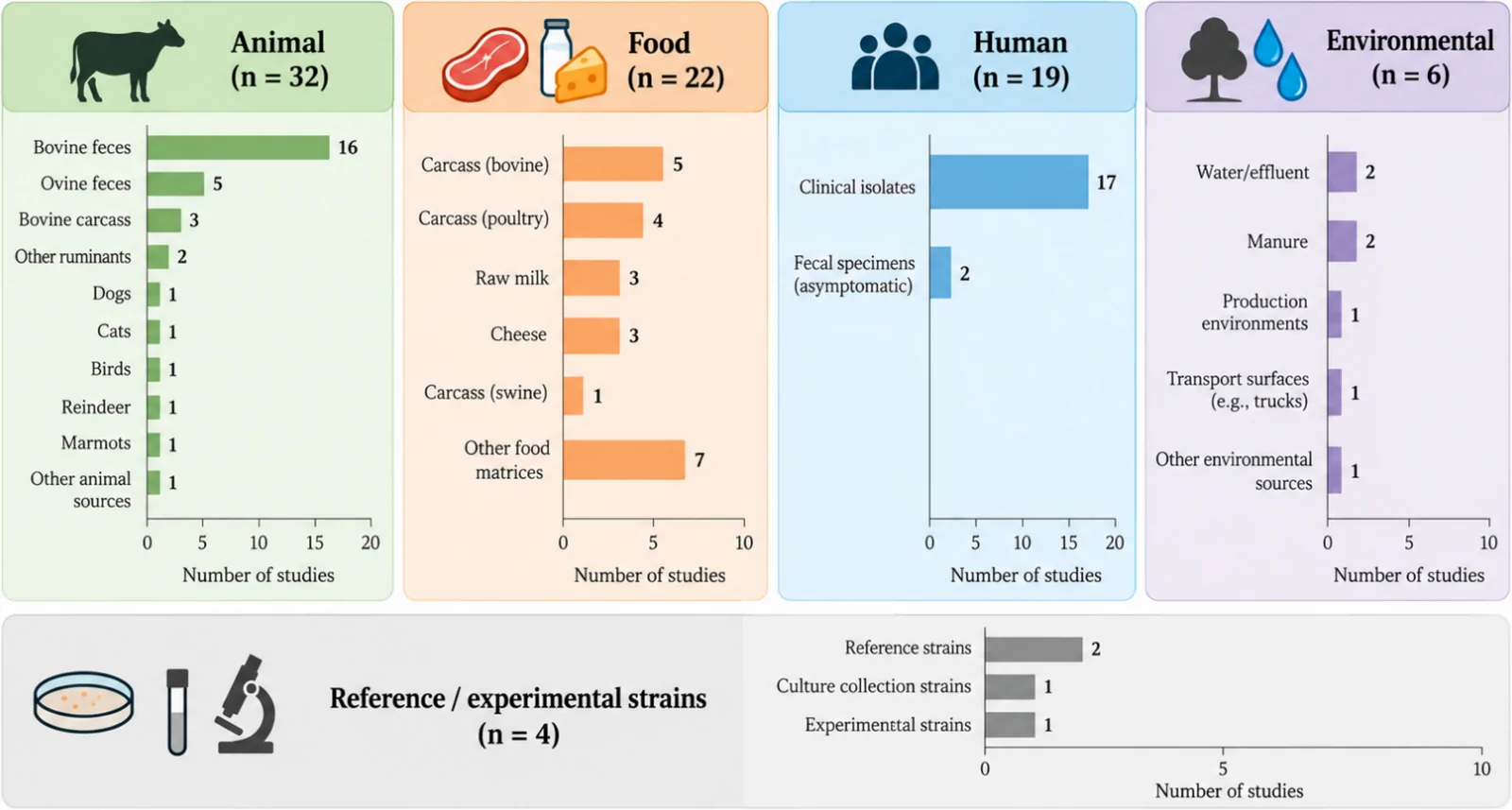
Distribution of major source categories and specific sample types reported in the studies included in the review of Shiga toxin-producing Escherichia coli (STEC). Major source categories and their corresponding subcategories are not mutually exclusive. Individual studies could include multiple sources or matrices and could therefore contribute to more than one subcategory within the same major source category. Consequently, the sum of the subcategories may exceed the total number of studies represented in the corresponding major category. Frequencies represent the number of studies reporting each source or sample type and should not be summed to estimate the total number of included studies

The included studies investigated STEC across a wide range of human, animal, food, and environmental sources. Because several studies included more than one source or matrix, the major source categories and their corresponding sample-type subcategories were not mutually exclusive. Individual studies could therefore contribute to multiple categories or subcategories. Animal sources were represented in 32 studies, food sources in 22 studies, human sources in 19 studies, and environmental sources in 6 studies. In addition, 4 studies included reference strains, culture collection strains, or strains used primarily in experimental settings (Fig. 2). Therefore, these frequencies represent the number of studies in which each source category was represented, and the frequencies of individual subcategories should not be summed to obtain the total number of studies within a major category or the total number of included studies.

Within these major source categories, specific sample types were identified and organized accordingly. Animal-related investigations included cattle, sheep, buffaloes, dogs, cats, birds, reindeer, marmots, and other animal sources, whereas food-related studies included carcasses, meat, raw milk, dairy products, and other food matrices. Human sources comprised clinical isolates and fecal specimens from symptomatic or asymptomatic individuals, while environmental investigations included water, wastewater, manure, and production- or outbreak-associated environments. This hierarchical classification of major source categories and their respective sample types is presented in Fig. 2.

The included literature also comprised heterogeneous study categories, including epidemiological/observational, genomic, surveillance, outbreak-related, experimental, mechanistic, and methodological investigations. These categories were considered separately during the descriptive synthesis according to their objectives, sampling frameworks, and analytical units. Experimental, mechanistic, and methodological studies without an epidemiological sampling framework were not used to infer STEC occurrence or prevalence.

### Detection methods, serotyping, and molecular typing

The studies included in this review employed a variety of laboratory approaches for the detection, identification, and molecular characterization of Shiga toxin-producing *Escherichia coli* (STEC). These approaches included conventional and quantitative PCR-based assays, serotyping or genoserotyping, multilocus sequence typing (MLST), whole-genome sequencing (WGS), single-nucleotide polymorphism (SNP)-based analyses, and other molecular or genomic methods. The specific methodological combinations varied according to study design and objective. Culture-based isolation procedures were described in several of the included studies, including Carvalho et al. [13], Lopes et al. [34], and Lucatelli et al. [37]. However, culture-based procedures were not systematically extracted as a standardized methodological variable across all included studies. Therefore, their overall frequency could not be reliably quantified. Biochemical identification procedures were likewise not systematically reported in the standardized methodological information extracted in Table 1 and were therefore not quantified.

PCR was the most frequently reported molecular method, being used in 47 of the 55 included studies (85.45%). PCR was primarily used to detect genes associated with Shiga toxin production, particularly *stx1* and *stx2*, although the specific molecular targets varied among studies. This percentage represents the proportion of studies reporting the use of PCR and not the proportion of samples or isolates tested by this method. Differences in target genes, assay formats, primer sets, and confirmation procedures limited direct methodological comparisons across studies.

Methods used for *E. coli* confirmation and serotype/serogroup characterization varied among the included studies. When explicitly reported, strain identification and characterization involved culture-based methods, PCR-based detection or genoserotyping, and sequencing-based approaches [26, 28, 55]. PCR-based assays were used for the detection of *stx* genes and other genetic targets, whereas genomic approaches enabled in silico characterization of serotype/serogroup and other molecular features [26, 28]. However, the specific procedures used for *E. coli* confirmation and serotype characterization were not consistently reported or extracted across all studies, preventing reliable study-level quantification of each individual approach.

Regarding serotype/serogroup characterization, O157 STEC was reported in 25 of the 55 included studies (45.45%), whereas non-O157 STEC was reported in 30 studies (54.55%). These categories were not mutually exclusive, as individual studies could report both O157 and non-O157 strains. Within the non-O157 category, some studies identified specific serogroups or serotypes, whereas others reported isolates only as non-O157 without further serotype characterization. Therefore, the term “non-O157” was used in this review as an overarching descriptive category and does not imply that complete serotype information was available for all isolates classified within this group. These percentages represent study-level reporting frequencies and should not be interpreted as the proportion of all analyzed isolates belonging to each serogroup. Carvalho et al. [13] was retained in the qualitative dataset as a study reporting detection of *E. coli* O157 in Minas Frescal cheese,however, because the original study did not demonstrate the presence of stx genes, its findings were not considered evidence of STEC and were therefore excluded from the STEC-specific serotype reporting count. Serotype/serogroup information was not reported in 16 studies (29.09%).

For genetic characterization, different molecular typing approaches were employed. MLST was reported in 15 studies (27.27%), allowing the identification of substantial sequence-type diversity among the strains analyzed. Multiple sequence types were identified across the retrieved studies, including ST11, ST21, ST16, ST17, ST443, ST677, and several other STs reported in individual investigations. ST11 was reported in nine studies; however, this study-level count includes studies in which ST11 was reported for comparator strains as well as studies in which it was assigned to isolates investigated as part of the study. Therefore, the nine-study frequency should not be interpreted as the number of studies investigating ST11-positive primary isolates or as evidence of the predominance of ST11 among the STEC isolates represented in the review. In addition, because ST11 was explicitly included in the original search strategy, its representation within the retrieved literature may have been influenced by search-related enrichment. Because sequence typing was not performed or reported uniformly across the included studies, frequencies of individual STs were interpreted as reporting patterns within the retrieved literature rather than frequencies of occurrence among STEC isolates or among studies evaluating sequence type. WGS was reported in 19 studies (34.55%), enabling detailed genomic characterization of STEC strains, including virulence determinants, sequence types, plasmids, antimicrobial resistance determinants, and phylogenetic relationships. SNP-based analyses were reported in five studies (9.09%) and were primarily used to investigate genomic relatedness and phylogenetic relationships among isolates. These percentages represent study-level frequencies of methodological reporting rather than the proportion of isolates analyzed using each approach.

Overall, the included literature demonstrated the use of both targeted molecular methods and higher-resolution genomic approaches for STEC characterization. However, because the included studies differed in sampling strategies, matrices, laboratory protocols, study objectives, and reporting practices, the frequencies presented in this section should be interpreted as descriptive study-level findings rather than pooled estimates of method performance, serotype distribution, or molecular characteristics among all STEC isolates.

### Virulence genes

Building upon the results presented in the previous section regarding detection and typing methods, the predominant use of molecular techniques, particularly PCR and whole-genome sequencing (WGS), enabled the identification of a broad diversity of virulence determinants associated with Shiga toxin-producing *Escherichia coli* (STEC) strains in the included studies.

Shiga toxin-encoding genes were widely represented in the retrieved literature. Based on determinants explicitly reported as detected in Table 1, *stx2* was reported in 40 studies and *stx1* in 29 studies. Studies in which these genes were only described as investigated, without explicit evidence of detection, as well as those reporting them as not detected (ND), were not counted as positive reports. These values therefore describe patterns of explicit reporting within the retrieved literature and should not be interpreted as frequencies of occurrence among studies specifically testing each gene or as the proportion of STEC isolates carrying these genes.

Shiga toxin subtyping was also reported across the included studies, revealing substantial subtype diversity. *stx2a* was explicitly reported in 17 studies, *stx2c* in 11 studies, and *stx2d* in seven studies. Additional *stx2* subtypes included *stx2b*, *stx2e*, *stx2f*, *stx2g*, *stx2h*, *stx2j*, *stx2m*, *stx2n*, and *stx2o*, with *stx2f* reported in four studies and *stx2h* in two. Regarding *stx1* subtypes, variants including *stx1a*, *stx1c*, *stx1d*, and *stx1e* were identified across the included studies. Because subtype testing was not performed or reported uniformly across studies, these counts represent reporting patterns within the retrieved literature rather than frequencies among studies specifically performing *stx* subtyping.

The distribution of Shiga toxin subtypes also varied according to the sources investigated. Subtypes including *stx2a*, *stx2c*, and *stx2d* were reported in studies involving human, animal, and food-derived STEC, whereas some other subtypes were reported in more restricted study populations or sources. However, because individual studies frequently included multiple isolate origins and subtype reporting was heterogeneous, source-specific frequencies were not pooled across studies. Therefore, relationships between stx subtype and isolate origin were interpreted descriptively rather than as quantitative associations.

Among genes associated with bacterial adhesion, eae, which encodes the adhesin intimin, was explicitly reported as detected in 20 studies. Additional genes involved in adhesion and colonization were also described, including *tir, espA, espB, espD, espF, espG, espJ, cif, efa1, lpfA, iha, hra, paa,* and *tccP.* Genes associated with additional toxins and plasmid-related virulence factors were also identified. The *ehxA* gene was explicitly reported as detected in 12 studies, whereas saa was reported in eight studies. *subA* was reported in three studies and *aggR* in one study. Studies in which these determinants were described only as investigated or evaluated, without explicit evidence of detection, were not included in these counts. Accordingly, these values represent study-level reporting patterns and should not be interpreted as frequencies among studies that specifically tested each determinant or as prevalence among STEC isolates.

Other virulence genes and pathogenicity-associated factors were reported across individual studies, including *astA*, *bfpA*, *cdt*, *cnf1*, *cnf2*, *hlyA*, *katP*, *espP*, *stcE*, *etpD*, *toxB*, *sab*, *pagC-like*, *hes*, *lesP*, and *ag43*, as well as genes related to the type III secretion system (T3SS). In addition, genes associated with plasmid features, colonization factors, and genetic markers used for molecular characterization included *iss*, *ompA*, *ompT*, *chuA*, *yjaA*, *terC*, *kpsMII_K5*, *traT*, *mchB*, *mchC*, and *mchF*. Housekeeping genes used in molecular typing schemes, such as *adk*, *fumC*, *gyrB*, *icd*, *mdh*, *purA*, and *recA*, were also reported; however, these genes were considered molecular typing markers rather than virulence determinants. Because these factors were reported inconsistently across studies, their frequencies were not individually quantified.

Virulence-determinant information was classified as not reported (NR) in some studies, while other studies explicitly reported determinants as not detected (ND) or described particular genes only as investigated. These categories were not treated equivalently: NR was not interpreted as evidence that a determinant had been tested, and genes reported only as investigated were not counted as detected. Overall, the included studies reported a broad diversity of virulence determinants in STEC strains, with frequent study-level reporting of *stx1*, *stx2*, and *eae*, as well as multiple Shiga toxin subtypes and additional factors associated with bacterial adhesion, colonization, toxin production, plasmid biology, and pathogenicity. These findings characterize reporting patterns in the retrieved literature and do not constitute estimates of the occurrence or prevalence of individual virulence determinants.

### Plasmids and pathogenicity islands

The analysis of the studies included in this review also allowed the identification of mobile genetic elements associated with the virulence of Shiga toxin-producing *Escherichia coli* (STEC) strains, including plasmids and pathogenicity islands. However, these genetic elements were not explicitly reported in most of the included studies, and absence of reporting was not interpreted as evidence that they had been investigated and found to be absent.

Information on plasmids or other mobile genetic elements was explicitly reported in 18 of the 55 included studies (32.7%), whereas 36 studies (65.5%) did not report this information and one study (1.8%) did not test these elements. When methodological information was available, these elements were characterized using molecular and genomic approaches, including PCR-based analyses, plasmid profiling, WGS, and plasmid sequencing and assembly. Among the reported mobile genetic elements, several studies described Shiga toxin-encoding bacteriophages or prophages, including *stx1*, *stx2*, *stx2a*, *stx2f*, and *stx2h* associated phages. Plasmid-related findings included pO157, a pO113-like plasmid, IncFIB and IncFII replicons, as well as other strain-specific plasmids and plasmid profiles. Some studies additionally reported virulence determinants associated with plasmids; these were distinguished from direct identification or characterization of the plasmids themselves.

Because the approaches and level of characterization varied substantially among studies, these elements were summarized descriptively rather than pooled by plasmid type**.** Because individual plasmids and replicons were not assessed across all 18 studies reporting mobile genetic elements, element-specific proportions were not calculated using 18 studies as a common denominator. Instead, individual elements were described according to their reporting in the retrieved literature, and absence of an element from a study report was not interpreted as a negative result unless the original study explicitly indicated that it had been evaluated and not detected. For example, Souza et al. reported one or more plasmids in 95% of the 38 STEC strains analyzed. Detailed plasmid profiling was reported for 36 strains, including 18/36 (50.0%) carrying a single approximately 90-kb plasmid.

Regarding pathogenicity islands and pathogenicity-associated genomic regions, information was explicitly reported or evaluated in 13 of the 55 studies (23.6%), while 38 studies (69.1%) did not report this information, three studies (5.5%) classified it as not applicable, and one study (1.8%) did not test these elements. Importantly, the studies grouped under this category did not necessarily characterize complete pathogenicity islands. For LEE, the reported analyses varied from explicit characterization of the presence or absence of the island to the detection of individual LEE-associated genes or type III secretion system effectors. Therefore, detection of eae or another LEE-associated gene was not interpreted as confirmation of the complete LEE island unless the original study explicitly characterized the island or its presence/absence. Two studies explicitly reported LEE-negative strains. Studies reporting only LEE-associated genes or effectors were retained as evidence of the corresponding genetic determinants but were not considered equivalent to studies that characterized the complete LEE region. Other pathogenicity-associated regions included OI-57, OI-122, and the locus of adhesion and autoaggregation (LAA). Michelacci et al., for example, evaluated LEE, OI-57, and OI-122, whereas other studies evaluated individual LEE-associated genes or effectors. Because the 13 studies did not uniformly investigate the same pathogenicity islands or associated loci, a common denominator of 13 was not applied to individual islands or associated genes.

Overall, the findings indicate that plasmids, bacteriophages/prophages, and pathogenicity islands were less consistently reported across the retrieved literature than the principal virulence determinants described in the previous section. The heterogeneity in the genetic elements investigated and in the methods used for their characterization precluded direct comparisons of their frequencies across studies. Therefore, these findings should be interpreted as study-level reporting patterns rather than estimates of the prevalence or comparative frequency of specific mobile genetic elements or pathogenicity islands among STEC isolates.

### Clinical manifestations associated with STEC infections

In addition to the microbiological and molecular characterization of Shiga toxin-producing *Escherichia coli* (STEC) strains, a subset of the studies included in this review also reported information on clinical manifestations associated with the analyzed isolates. However, most studies did not focus on this aspect, and clinical manifestations were not reported (NR), not tested (NT), or considered not applicable (NA) in a substantial proportion of the included studies. Clinical information was therefore interpreted according to the analytical unit and context of each original study rather than aggregated as estimates of symptom frequency across the retrieved literature.

Among studies involving human clinical isolates or cases, reported manifestations included diarrhea, bloody diarrhea, abdominal pain or cramps, vomiting, fever, hemolytic uremic syndrome (HUS), hemorrhagic colitis, and other clinical outcomes. Where the original publication provided an explicit denominator, this information was retained at the study level. For example, Den Ouden et al. reported HUS in 7/40 cases with available clinical information (17.5%), Baba et al. reported bloody diarrhea in 28/38 symptomatic patients (74%) and HUS in 1/38 (2.6%), Bai et al. reported diarrhea in 2/3 cases and asymptomatic carriage in 1/3, and Underwood et al. reported HUS in approximately 22% of 93 outbreak cases. These study-specific proportions were not pooled because the populations, clinical contexts, case definitions, and reporting approaches differed substantially among studies.

Some studies also included isolates associated with particular clinical conditions without providing a denominator suitable for estimating the frequency of those outcomes. In such cases, the clinical information was retained only as contextual information regarding the origin or characterization of the isolates and was not interpreted as an estimate of disease occurrence.

Animal-associated studies more commonly described the health status of the sampled animals, including healthy or asymptomatic animals and animals with diarrhea, rather than establishing clinical disease attributable specifically to STEC. Accordingly, animal health status was distinguished from clinical outcomes directly attributed to STEC infection. Experimental studies in which clinical manifestations were not relevant to the study design were classified as not applicable (NA).

\Overall, the clinical information available in the retrieved literature was limited and heterogeneous. Reported outcomes ranged from enteric manifestations, such as diarrhea and bloody diarrhea, to severe clinical presentations including HUS. Because clinical data were not collected or reported uniformly and because the analytical units differed among studies, these findings were interpreted descriptively and were not used to estimate the overall frequency or prevalence of individual clinical outcomes.

### Antimicrobial resistance

Antimicrobial resistance (AMR) was investigated or reported in a subset of the 55 included studies using phenotypic antimicrobial susceptibility testing, detection of antimicrobial resistance determinants, or both. However, the analytical populations differed among studies. While some studies provided resistance data specifically for STEC isolates, others reported AMR results for broader *E. coli* populations without providing separate estimates for the STEC subset. Accordingly, only findings explicitly attributable to STEC isolates were interpreted as STEC-specific in the present review. Resistance findings reported for broader *E. coli* populations were retained as contextual information but were not used to characterize antimicrobial resistance in STEC. In the remaining studies, antimicrobial resistance was not reported (NR), not tested (NT), or considered not applicable (NA).

Phenotypic antimicrobial susceptibility was evaluated in a subset of the included studies, although the antimicrobial panels, testing methods, and analytical populations differed substantially among them. Among studies providing results specifically attributable to STEC or stx-positive isolates, resistance was reported to antimicrobials from different classes, including tetracyclines, aminoglycosides, quinolones, sulfonamides, and other agents. Because the antimicrobial panels were not standardized and some studies reported susceptibility results only for broader *E. coli* populations, no study-level ranking of individual antimicrobial resistance frequencies was performed. Results from broader *E. coli* populations were not interpreted as STEC-specific resistance findings.

Among studies providing phenotypic resistance results specifically attributable to STEC isolates, Arrais et al. [1] reported resistance to at least one antimicrobial in 57/59 bovine isolates (96.6%) and 42/47 ovine isolates (89.4%), with MDR reported in 40/59 (67.8%) and 28/47 (59.6%), respectively. Ferreira et al. [23] reported resistance to at least one antimicrobial in 75/90 STEC isolates (83.3%), resistance to two antimicrobials in 11/90 (12.2%), and resistance to three or more antimicrobials in 6/90 (6.7%). Mussio et al. [49] reported no phenotypic resistance among the 39 STEC isolates by disk diffusion, although one isolate showed resistance to tetracycline and minocycline when evaluated by MicroScan. These isolate-level results refer exclusively to the populations and methods evaluated in the respective original studies and were not pooled or directly compared across studies.

Deng et al. [21] evaluated phenotypic antimicrobial resistance in the complete *E. coli* population analyzed (*n* = 178), which included STEC, ETEC, hybrid STEC/ETEC, and unclassified isolates. Because phenotypic resistance and MDR results were not reported separately for the STEC subset, these proportions were retained in Table 1 as contextual information but were not included in the STEC-specific interpretation of phenotypic antimicrobial resistance. The study additionally reported the distribution of selected antimicrobial resistance genes according to pathotype,these genotypic data were interpreted separately according to the analytical groups defined in the original study. Similarly, antimicrobial susceptibility results reported for broader E. coli populations in other studies were not attributed to STEC unless a STEC-specific denominator was explicitly provided.

Genotypic antimicrobial resistance was investigated in a subset of the included studies using genomic or molecular approaches. Among studies providing results specifically attributable to STEC isolates, acquired resistance determinants associated with several antimicrobial classes were identified, including aminoglycosides, tetracyclines, sulfonamides/trimethoprim, β-lactams, quinolones, macrolides, phenicols, fosfomycin, and colistin. The specific determinants and their distribution varied considerably among studies and isolate populations. Because the genomic approaches, resistance databases, analytical populations, and determinants investigated were heterogeneous, study-level frequencies of antimicrobial resistance classes were not calculated. Genotypic findings reported only for broader E. coli populations were not interpreted as STEC-specific resistance data.

At the individual determinant level, STEC-specific studies identified acquired resistance genes including *tetA/tetB*, *blaTEM-1/blaTEM-1 B*, *qnrB19*, *sul2, aph(3″)-Ib, aph(6)-Id*, *aph(3′)-Ia*, *fosA7*, *mdf(A)*, and *mcr-1*, among other determinants. Lopes et al. [35], for example, detected *mcr-1* in 2/35 STEC isolates (5.7%), whereas Baba et al. [5] detected acquired antimicrobial resistance genes in 18/65 STEC isolates (28%). Gonzalez-Escalona and Kase [28] identified acquired resistance genes in 33/276 STEC isolates (12%). Lucatelli et al. [37] detected no acquired antimicrobial resistance genes in the STEC O157:H7 ST11 isolate evaluated, which was phenotypically susceptible to the antimicrobials tested, although chromosomal mutations were identified in *gyrA*, *gyrB*, *parC*, *parE*, *pmrA*, *pmrB*, and *folP*. Mussio et al. [49] also identified resistance-associated genes despite the limited phenotypic resistance observed, illustrating that genotypic and phenotypic findings were not necessarily equivalent across the included studies.

The relationship between genotypic and phenotypic resistance varied among studies in which both types of information were available. Bai et al. [7] detected the macrolide resistance-associated gene *mdf(A)* in all three *stx2m*-STEC isolates analyzed, while all three remained phenotypically susceptible to the antimicrobials tested, including azithromycin. Mussio et al. [49] detected *tetB*, *sul2*, *aph(3″)-Ib*, and *aph(6)-Id* in one STEC isolate, with phenotypic resistance to tetracycline and minocycline subsequently confirmed by MicroScan, whereas fosA7 was detected in another isolate without corresponding phenotypic resistance to fosfomycin. These findings illustrate that the detection of resistance determinants does not necessarily correspond directly to phenotypic resistance and that genotype–phenotype relationships should be interpreted within the analytical framework of each individual study.

Antimicrobial resistance findings were reported in studies involving animal, food, and human sources, including studies that simultaneously investigated isolates from more than one of these compartments. For example, Galarce et al. [26] investigated cattle, beef, and human isolates, whereas other studies evaluated AMR in animal or food-associated STEC populations. No AMR data were available for the environmental studies represented in the current dataset. Because source categories, antimicrobial panels, susceptibility testing methods, and genomic approaches varied substantially among studies, no formal comparisons of resistance frequencies according to isolate origin were performed.

Overall, the included studies demonstrated heterogeneous phenotypic and genotypic AMR findings among STEC isolates. Differences in study populations, antimicrobial panels, susceptibility-testing methods, genomic approaches, and reporting strategies precluded pooled estimates or direct comparisons of resistance frequencies across studies. Therefore, AMR findings were interpreted within the analytical population and methodological context of each original study.

## Discussion

### Geographic distribution and epidemiological aspects

According to the studies included in this review, the available evidence on STEC showed broad geographic coverage, although the included literature was unevenly distributed, with a greater representation of studies from the Americas, particularly Brazil and the United States. This pattern is reflected in studies assessing the occurrence of the pathogen across different matrices, including bovine and ovine feces, raw milk, dairy products, and production environments, highlighting the relevance of livestock production systems in the maintenance and dissemination of STEC [1, 24, 53, 63].

Based on the analyzed data, the higher concentration of studies in these regions does not necessarily indicate a higher prevalence of the pathogen and should primarily be interpreted as greater representation of these countries within the literature retrieved by the present search strategy. Differences in surveillance capacity, laboratory infrastructure, research activity, and the application of molecular methods may also contribute to variation in STEC detection and reporting across settings. Molecular approaches, including PCR and whole-genome sequencing, have enabled detailed detection and characterization of STEC across food, animal, human, and environmental sources [28, 43, 61].

The comparatively high representation of Brazilian studies may also reflect characteristics of the country’s livestock and food production sectors. Brazil has a major role in global animal protein production and is among the world’s largest beef producers and exporters [62]. This economic and productive relevance, particularly within the cattle and beef sectors, may contribute to sustained scientific interest in STEC surveillance and characterization in cattle, carcasses, meat products, and related production settings. Together with established microbiological research and surveillance activities, these factors may partly explain the comparatively large number of Brazilian studies identified in the present review. However, this interpretation should be considered a possible explanation for the representation of Brazil in the retrieved literature rather than a causal relationship demonstrated by the review data.

The studies included in this review also demonstrate that STEC occurs across multiple interfaces involving animals, food products, and the environment. The presence of the pathogen in bovine carcasses, food products, transport-associated environments, and animal **sources** highlights the complexity of its circulation across different components of the food production chain [17, 26, 31, 49]. Environmental and production-associated matrices, including water, feces, insects, and organic residues, were also represented among the included studies [14, 25, 39, 40, 57].

Furthermore, differences in epidemiological surveillance systems, the application of diagnostic methods, and reporting requirements across countries may influence STEC detection and the availability of epidemiological data. Consequently, comparisons among countries should be interpreted cautiously, particularly because the studies included in this review differed substantially in design, sampling strategy, matrices, and molecular approaches [5, 20, 55].

Overall, the findings of this review support a geographically diverse but unevenly represented evidence base for STEC and demonstrate its detection across animal, food, human, and environmental contexts. Rather than providing estimates of the global burden or country-level prevalence of STEC, the present findings characterize the geographic and epidemiological patterns represented in the studies identified by the review strategy. This scenario underscores the need for integrated approaches to pathogen monitoring, particularly considering its relevance at the interface between animal production, food systems, and human health.

### Animal reservoirs and one health

According to the studies included in this review, food-producing animals, particularly cattle and sheep, constitute important reservoirs of STEC, playing a central role in the maintenance and dissemination of the pathogen. Several studies identified the presence of STEC in feces, milk, and carcasses from these animals, highlighting their epidemiological importance within the food production chain [1, 23, 53, 63].

Based on the analyzed data, the occurrence of STEC in cattle is particularly relevant, as these animals can act as asymptomatic reservoirs and shed the microorganism in their feces [24, 51]. In cattle, the terminal rectum, particularly the rectoanal junction, is recognized as an important site of STEC O157:H7 colonization, establishing a direct relationship between intestinal colonization and subsequent fecal shedding [44, 56]. Fecal shedding can vary considerably among animals, and a relatively small proportion of cattle may excrete high concentrations of the pathogen, a phenomenon commonly referred to as “super-shedding” [16, 41]. Such animals may contribute disproportionately to transmission within cattle populations and to contamination of the production environment.

Fecal shedding also provides an important route for STEC contamination of soil, water, equipment, surfaces, and other components of the production environment. During slaughter and processing, fecal contamination can facilitate the transfer of STEC to hides, equipment, processing surfaces, and carcasses, creating opportunities for cross-contamination and subsequent entry of the pathogen into the food chain [49, 51]. These pathways highlight the importance of hygienic and control measures throughout primary production, transport, slaughter, and food processing to minimize fecal contamination and cross-contamination.

As observed in the studies included in this review, the presence of STEC is not limited to food-producing animals but has also been identified in other species, including birds, dogs, cats, and buffaloes, as well as in insects and environments associated with animal production [14, 18, 19, 32]. These findings indicate that the circulation of the pathogen occurs across multiple hosts and environmental niches, reinforcing the complexity of its epidemiology.

According to the analyzed studies, the detection of STEC in different matrices, including food, water, environmental samples, and transport surfaces, highlights the potential connections among animals, food, and the environment in the circulation of the pathogen [17, 39, 40]. The detection of STEC in dairy products, such as cheese and raw milk, further highlights the importance of appropriate hygienic practices during food production, handling, and processing [12, 13, 46].

Another important aspect concerns the interface between animal and human health, as studies evaluating clinical isolates and isolates from animal and food sources have reported shared serotypes, sequence types, and/or virulence-associated characteristics across these sources [5, 20, 28]. This supports the relevance of STEC at the animal–food–human interface and reinforces the importance of foodborne transmission in its epidemiology.

Overall, the findings from the included studies support the importance of an integrated approach to STEC epidemiology that considers the interconnected roles of animals, food, the environment, and humans. In this context, the One Health approach provides an appropriate framework for integrated surveillance and the development of control and prevention strategies, particularly with regard to food safety and the reduction of infection risks associated with the consumption of contaminated products.

### Genetic diversity, serotypes, and sequence types

According to the studies included in this review, the genetic diversity of STEC represents one of the most relevant findings for understanding its epidemiology, as this group comprises strains with distinct serotypes, virulence profiles, and genetic backgrounds. Although the O157:H7 serotype remains one of the most recognized in the literature, mainly due to its historical association with outbreaks and severe clinical outcomes, the data from this review indicate that non-O157 serotypes also play a prominent role, reinforcing the need for a broader approach in STEC surveillance and laboratory characterization [24, 28, 31, 37].

Based on the analyzed data, the persistence of O157:H7 as an epidemiological reference does not imply exclusivity in terms of clinical or public health relevance. Studies conducted in Brazil and other countries reported O157:H7 in cattle and food-related sources, including beef and dairy products, supporting its continued relevance in animal reservoirs and foods of animal origin [13, 24, 25, 37, 66]. However, studies employing broader molecular and genomic characterization also identified considerable diversity among non-O157 serotypes and their virulence-associated genetic profiles [26, 43, 54].

As observed in genomic and molecular characterization studies, expanding the focus to non-O157 strains has broadened the characterization of STEC diversity. Kobayashi et al. [31], through virulence gene profiling and population analysis, showed that virulence-associated determinants occur across a heterogeneous set of STEC serotypes and genetic backgrounds. Similarly, Gonzalez-Escalona and Kase [28] identified a broad diversity of *stx*-positive strains in FDA-regulated foods, highlighting that STEC characterization in food should not be restricted to O157. These findings are consistent with Galarce et al. [26], who, by analyzing isolates from the animal–food–human interface in South America, identified diverse STEC genomic profiles across these sources, reinforcing the value of integrating molecular epidemiology with public health surveillance.

According to clinical and genomic studies included in this review, non-O157 serotypes were also represented among human, animal, and food-associated isolates and displayed diverse combinations of virulence-associated determinants. Notably, several epidemiologically relevant non-O157 serotypes were identified in the included literature, including O26:H11, O45:H2, O103:H2 and O103:H25, O111:H2 and O111:NM, O121:H19, and different O145 serotypes [10, 55, 65]. These serotypes were represented across studies involving human, animal, and food-associated isolates, illustrating that the serotypic diversity observed in the reviewed literature extended beyond O157:H7. Other studies, including Baba et al. [5], Bai et al. [7], and Den Ouden et al. [20], further demonstrated genetically diverse non-O157 STEC among human isolates with variation in their virulence-associated genetic profiles. Collectively, these findings support the importance of laboratory approaches capable of detecting and characterizing STEC beyond a limited set of traditional serogroups and demonstrate that the serotypic diversity observed in this review reflects underlying genetic heterogeneity relevant to molecular characterization, epidemiological investigation, and surveillance.

MLST-based characterization further demonstrated the genetic diversity of STEC represented in the retrieved literature. Among the sequence types reported, ST11 was reported in nine of the 55 included studies (16.36%); however, this study-level count includes at least one study in which ST11 was reported in an O157:H7 comparator strain rather than in the primary isolates investigated. Therefore, this frequency should not be interpreted as the proportion of studies investigating ST11-positive primary isolates or as evidence of its predominance among STEC lineages. ST21 was reported in four studies (7.27%), while ST16 and ST17 were each reported in two studies (3.64%), and ST443 and ST677 were each reported in one study (1.82%). These findings demonstrate that the STEC strains characterized in the included literature comprised multiple genetic backgrounds rather than being dominated by a single sequence type. The representation of ST11 should also be interpreted cautiously because it was explicitly included in the original search strategy and may therefore have been subject to search-related enrichment. Lucatelli et al. [37], for example, identified an O157:H7 ST11 isolate in ground beef in Brazil, providing a food-associated example of this lineage, but this individual finding should not be interpreted as evidence of its predominance or epidemiological importance on its own.

When comparing studies focused on O157 with those dedicated to broader STEC characterization, the included literature illustrates the complementary information provided by serotyping and higher-resolution molecular approaches. Studies centered on O157, such as those by Freitas Filho et al. [24], Frozi et al. [25], and Lucatelli et al. [37], documented this serotype in animal and food-associated sources. In contrast, Carvalho et al. [13] detected *E. coli* O157 in Minas Frescal cheese using serological and PCR-based O157/H7 markers but did not demonstrate the presence of *stx* genes,therefore, this study was not considered evidence of STEC in the STEC-specific synthesis. Studies based on molecular subtyping, comparative genomics, and molecular surveillance, such as Scheutz et al. [54], Michelacci et al. [43], Galarce et al. [26], Lindsey et al. [33], and Projahn et al. [52], revealed broader genetic and serotypic diversity among STEC strains. Together, these findings indicate that O157 remains relevant but does not encompass the full diversity represented by STEC.

Furthermore, the genetic variability observed across studies suggests that STEC characterization benefits from approaches that extend beyond serological classification. Studies such as Michelacci et al. [43], Steyert et al. [59], and Lorenz et al. [36] reported differences in virulence-associated genes, plasmids, pathogenicity-associated regions, and other genomic characteristics among STEC strains. Therefore, strains sharing the STEC classification may differ substantially in their genetic composition, supporting the use of higher-resolution molecular approaches for strain characterization and epidemiological surveillance.

Overall, the data indicate that the genetic and serotypic diversity of STEC represents a central axis for interpreting the heterogeneity of strains identified across human, animal, food, and environmental sources. Although O157:H7 remains epidemiologically relevant, the diversity of non-O157 serotypes and sequence types observed across the included studies indicates that STEC cannot be adequately characterized on the basis of a single serotype or genetic lineage. Collectively, the analyzed studies support the integration of serotyping, *stx* subtyping, and genomic approaches for more comprehensive STEC characterization and surveillance [26, 28, 37, 54].

### Virulence genes and molecular mechanisms of pathogenicity in STEC

According to the studies included in this review, STEC strains exhibited diverse combinations of virulence determinants, including *stx1*, *stx2*, and their subtypes, as well as other determinants such as *eae*, *ehxA*, *saa*, and *subA*. Both *stx1* and *stx2* were widely represented in the retrieved literature, although their evaluation and reporting were not uniform across studies. Therefore, differences in the number of studies reporting these genes were interpreted as patterns within the retrieved literature rather than as evidence of differences in their frequency of occurrence among STEC strains [1, 21, 23, 51]. More importantly, the heterogeneous combinations of these determinants indicate that STEC virulence cannot be interpreted as a uniform characteristic of the pathotype. Instead, pathogenic potential emerges from the combination of toxin subtype, adhesion-associated determinants, mobile genetic elements, and the broader genomic background of individual strains.

The broad representation of *stx2* in the retrieved literature is relevant in the context of previous evidence showing that specific *stx2* subtypes may differ in their association with STEC virulence and clinical outcomes. Studies evaluating clinical and food-derived isolates show that strains harboring certain *stx2* subtypes, particularly when combined with other virulence factors, may be associated with severe clinical outcomes, including hemolytic uremic syndrome (HUS). In this context, the mere detection of the gene is not sufficient to estimate the pathogenic potential of a strain, and the toxin subtype and broader genetic background should also be considered [5, 28, 31]. This distinction has epidemiological relevance because surveillance approaches based exclusively on the detection of *stx* may identify STEC but provide limited information for differentiating strains with distinct virulence profiles.

As observed in subtyping studies, the diversity of *stx* variants represents a central aspect in understanding STEC virulence. Studies such as Scheutz et al. [54] contributed to the standardization of *stx* subtype nomenclature, enabling the differentiation of variants with distinct biological and epidemiological characteristics. This distinction is particularly relevant for *stx2*, as subtypes such as *stx2a*, *stx2c*, and *stx2d* have been investigated in relation to severe clinical outcomes, whereas other subtypes may differ in their host distribution and clinical significance [54, 42]. Therefore, *stx* subtyping provides information beyond the detection of Shiga toxin genes and may contribute to molecular characterization, epidemiological investigation, and assessment of the pathogenic potential of STEC strains. More recent studies have further expanded the recognized diversity of *stx* subtypes, describing additional variants and highlighting the genetic diversity of *stx*-encoding elements [6, 33, 52]. The identification of uncommon or recently described subtypes also emphasizes the value of molecular methods capable of resolving toxin variants rather than treating stx1 and stx2 as homogeneous categories.

Within the present review, the diversity of *stx* subtypes was also evident at the study level. *stx2a* was explicitly reported in 17 studies, *stx2c* in 11 studies, and *stx2d* in seven studies, whereas *stx2f* was reported in four studies and *stx2h* in two studies. Additional subtypes, including *stx2b*, *stx2e*, *stx2g*, *stx2j*, *stx2m*, *stx2n*, and *stx2o*, were reported less consistently. These less frequently reported subtypes are considered uncommon only in the context of the retrieved literature and should not be interpreted as biologically rare variants in the broader STEC population. Because subtype testing was not performed uniformly and the number and type of isolates varied substantially among studies, these reporting frequencies cannot be used to estimate the prevalence of individual subtypes.

The available studies also indicate that stx subtype diversity extended across different isolation sources, although no quantitative source-specific association can be established from the present dataset. The more frequently reported subtypes *stx2a*, *stx2c*, and *stx2d* occurred in studies involving human, animal, and food-derived STEC. In food-derived isolates, Gonzalez-Escalona and Kase [28] identified *stx2a*, *stx2b, stx2c*, *stx2d*, *stx2d/e*, *stx2e*, and *stx2g*, among other *stx* variants. In animal-associated isolates, *stx2b* and *stx2c* were reported among reindeer-associated STEC, while *stx2a* was identified in a cattle-associated study. Human clinical studies provided examples of additional, less frequently reported subtypes: *stx2f* was identified among human fecal isolates, while *stx2j*, *stx2m*, *stx2n*, and *stx2o* were identified among human clinical isolates. These findings indicate that subtype diversity is distributed across multiple epidemiological contexts rather than being restricted to a single source. However, because several studies included more than one source and subtype detection was not standardized, these observations should be interpreted as descriptive examples rather than evidence of source-specific enrichment.

The occurrence of less frequently reported subtypes in human-associated studies is particularly relevant to interpretation of pathogenic potential. The study reporting *stx2f* involved human fecal specimens and documented diarrhea, bloody diarrhea, vomiting, fever, and abdominal pain, with HUS reported in seven of 40 cases for which clinical information was available. Similarly, *stx2m* was identified in human clinical isolates obtained from diarrheal patients and an asymptomatic carrier. However, these observations do not establish that the respective subtypes caused or determined the clinical outcomes observed. The available data do not consistently link each subtype to individual clinical manifestations, and the number of isolates characterized for each variant is limited. Therefore, the presence of an uncommon subtype should be interpreted as an indication of genetic diversity rather than as an independent marker of disease severity.

An important molecular feature of STEC is that *stx* genes are typically carried by Shiga toxin-encoding bacteriophages, which are frequently integrated into the bacterial chromosome as prophages. Consequently, Shiga toxin expression is closely linked to phage biology, particularly prophage induction and regulation of the lytic cycle. These bacteriophages also provide a mechanism for the horizontal transfer of *stx* genes between bacterial backgrounds, contributing to the genomic diversification and evolution of STEC virulence [50]. Thus, the phage-associated nature of stx provides a biological explanation for part of the heterogeneity observed among STEC strains: toxin production may depend not only on which stx subtype is present, but also on the genomic context, regulation, and biological behavior of the prophage carrying it. This phage-associated organization provides an important framework for understanding differences in toxin expression, regulation, and propagation among STEC strains.

Beyond the descriptive epidemiological studies, the experimental and mechanistic studies included as complementary evidence provided further information on the molecular mechanisms associated with *stx2* production and release, particularly those involving phage regulation. Atitkar et al. [3] demonstrated that phage-encoded lytic genes associated with *stx2* are not essential for virulence in murine models nor for toxin release into the bacterial supernatant, suggesting the existence of alternative pathways for *stx2* secretion. In a complementary study, Atitkar and Melton-Celsa [4] showed that variations in the regulatory region of the *stx2a* phage influence toxin localization and strain virulence, highlighting that certain clinical isolates are capable of producing *stx2* independently of the classical *RecA*-mediated system. Together, these observations help explain why detection of the same toxin gene or subtype does not necessarily imply equivalent toxin expression or biological behavior among strains. These experimental findings illustrate that the pathogenic potential associated with *stx2* may depend not only on gene presence but also on its phage-associated genetic context and regulation**.**

This interpretation is also relevant to horizontal gene transfer. Because stx-encoding phages can occur in different bacterial genomic backgrounds, acquisition, loss, or modification of these elements can contribute to the emergence of STEC lineages with new combinations of virulence characteristics [50, 55]. In epidemiological terms, this genomic plasticity complicates prediction of pathogenic potential based exclusively on serotype or a limited set of virulence markers and reinforces the contribution of genomic characterization to STEC surveillance [50].

Similarly, Steyert et al. [59] and Lorenz et al. [36] highlighted the role of the accessory genome and plasmids in the genetic diversity of STEC virulence profiles, indicating that strains classified within the same broad group may differ substantially in their virulence-associated genetic content. This variability is particularly relevant for LEE-negative STEC, because absence of the classical LEE-associated adhesion mechanism does not imply absence of other determinants capable of contributing to colonization or pathogenicity.

In addition to toxin genes, other virulence factors also play an important role in STEC pathogenesis. The presence of the *eae* gene, associated with the formation of attaching and effacing (A/E) lesions, was frequently reported among the included studies and occurred in different combinations with *stx* genes [31, 43]. However, detection of eae alone does not establish the presence of the complete locus of enterocyte effacement (LEE), as clarified in the revised Results. Studies differed in whether they characterized the LEE region itself or evaluated individual LEE-associated genes or effectors. On the other hand, genes such as *saa* and *subA* were reported in LEE-negative STEC strains in some studies, indicating that alternative adhesion and colonization mechanisms can occur in the absence of LEE [28, 51]. The presence of *ehxA*, often associated with virulence plasmids, further illustrates the diversity of additional virulence-associated determinants reported among STEC strains [36, 58]. Taken together, these findings support interpretation of STEC virulence as a multifactorial phenotype rather than one determined solely by the presence of Shiga toxin genes.

The different methodological approaches represented in the included literature provided complementary levels of information on STEC virulence. Studies based on PCR and genetic screening, such as those by Arrais et al. [1], Ferreira et al. [23], and Deng et al. [21], identified the occurrence and distribution of key virulence genes among the strains investigated. Genomic and functional studies, such as those by Atitkar et al. [3, 4], Lindsey et al. [33], and Michelacci et al. [43], provided additional information on genetic context, subtype diversity, regulation, and functional characteristics that cannot be inferred from gene detection alone. This methodological progression is epidemiologically important: targeted PCR remains useful for detection and surveillance, whereas genomic and functional approaches can resolve combinations of virulence determinants, mobile genetic elements, and regulatory contexts that may differentiate strains with otherwise similar molecular screening profiles.

Overall, the findings of this review highlight the substantial diversity of virulence-associated determinants reported among STEC strains. Rather than the predominance of any single determinant, the principal interpretative pattern emerging from these studies is the heterogeneity of virulence architectures represented among STEC. The diversity of *stx* subtypes across human, animal, and food-associated studies further illustrates that Shiga toxin characterization provides information beyond the binary detection of *stx1* or *stx2*. However, the present evidence does not support assigning a specific clinical risk to individual subtypes solely on the basis of their occurrence or reporting frequency. The presence of *stx* genes, the diversity of their subtypes, their phage-associated genetic context, and their combination with other virulence determinants contribute complementary information to STEC characterization.

### Clinical manifestations and virulence-associated factors in STEC

Clinical information was available for only a limited subset of the studies included in this review. Most studies did not provide detailed descriptions of clinical manifestations or clinical metadata linked to the characterized isolates, limiting the possibility of establishing direct genotype–phenotype relationships. Therefore, the clinical findings identified in this review should be interpreted descriptively rather than as evidence of consistent associations between specific virulence profiles and disease severity. This limitation is particularly relevant because the included studies differed in their analytical populations, which comprised individual isolates, clinical cases, outbreak populations, and animal or food-associated samples.

Among the studies that reported clinical information, manifestations included diarrhea, bloody diarrhea, abdominal pain or cramps, vomiting, fever, hemorrhagic colitis, and hemolytic uremic syndrome (HUS). Where explicit denominators were available, substantial variation was observed among individual studies. Den Ouden et al. [20] reported HUS in 7 of 40 cases with available clinical information (17.5%), whereas Baba et al. [5] reported bloody diarrhea in 28 of 38 symptomatic patients (74%) and HUS in 1 of 38 patients (2.6%). Bai et al. [7] reported diarrhea in two of three individuals and asymptomatic carriage in one of three. These findings illustrate the heterogeneity of the clinical populations represented in the review and were therefore not pooled or interpreted as estimates of clinical outcome frequency among STEC infections.

Some included studies provided molecular and clinical information within the same investigation, allowing a more contextual interpretation of pathogenic potential. Den Ouden et al. [20], for example, identified *stx2f*, *eae*, *astA*, *bfpA*, and *cdt* among non-O157 human fecal isolates and reported clinical manifestations including diarrhea, bloody diarrhea, vomiting, fever, abdominal pain, and HUS. Although the coexistence of these virulence-associated determinants and clinical outcomes is relevant, the available evidence does not support attributing disease severity to stx2f or to any individual determinant.

A similar pattern was observed for *stx2m*. Bai et al. [7] identified *stx2m*-STEC isolates from individuals with diarrhea as well as from an asymptomatic carrier. Although this observation demonstrates that the same toxin subtype may occur in different clinical contexts, the very small number of isolates prevents any inference regarding a specific clinical phenotype associated with *stx2m*.

Within the broader STEC literature, *stx2* and specific *stx2* subtypes have been associated with severe clinical outcomes, including HUS. However, this broader evidence should be distinguished from the findings of the present review. The studies included here provide examples of *stx* subtypes occurring in symptomatic and asymptomatic contexts, but the available clinical information is insufficient to quantify the contribution of individual subtypes to disease severity. Thus, stx subtype should be considered one component of pathogenic potential rather than an independent predictor of clinical outcome.

The diversity of virulence-associated factors beyond Shiga toxin also contributes to the complexity of STEC pathogenicity. The *eae* gene encodes intimin and is involved in the formation of attaching and effacing lesions, while LEE-negative strains may carry alternative adhesion-associated genes such as *saa* [2, 28, 31, 43, 59]. Within the included studies, Galarce et al. [26] detected several LEE-associated determinants, including *eae*, *tir*, *espA*, *espB*, *espJ*, *nleA*, *nleB*, and *nleC*, among isolates associated with patients, whereas *saa* was detected in five isolates obtained from asymptomatic food handlers. This contrast illustrates that virulence-associated genes may occur in both symptomatic and asymptomatic contexts and should therefore be interpreted within their host and genomic background.

Other determinants reported among the included studies included *ehxA*, associated with enterohemolysin production, and *subA*, encoding subtilase cytotoxin, as well as pathogenicity-associated regions such as LEE, OI-57, OI-122, and the locus of adhesion and autoaggregation (LAA) [26, 36, 40, 58]. Importantly, the detection of individual genes should not be interpreted as evidence of equivalent functional activity or clinical relevance across isolates. In particular, the presence of *eae* does not by itself establish the presence of the complete LEE pathogenicity island, as different studies evaluated either individual LEE-associated genes or broader genomic regions.

Several studies involving animal-origin isolates described apparently asymptomatic hosts, particularly cattle and sheep, consistent with the role of these animals in the maintenance and fecal shedding of STEC [8, 22, 24]. In contrast, Caliman and Marin [11] investigated *E. coli* isolates obtained from cats, including animals with urinary tract infection, but did not detect *stx1* or *stx2*. Therefore, this study does not provide evidence of STEC occurrence in the feline isolates and was not considered in the STEC-specific interpretation of animal reservoirs. In this context, asymptomatic carriage should be interpreted primarily as evidence of colonization, maintenance, and shedding rather than as a clinical manifestation. Fecal shedding may facilitate contamination of the farm environment, carcasses, and food products without evident clinical signs in the animal. Conversely, food-derived isolates represent evidence of contamination within the food chain and cannot be assigned a clinical phenotype based on their source alone.

The coexistence of symptomatic and asymptomatic contexts was also observed among human-associated isolates. Galarce et al. [26] investigated both symptomatic patients and asymptomatic food handlers, while Bai et al. [7] identified *stx2m*-STEC in both diarrheal patients and an asymptomatic carrier. These observations reinforce that the presence of STEC or a particular virulence determinant does not necessarily result in the same clinical expression across hosts. Host characteristics, bacterial genetic background, toxin subtype, regulatory mechanisms, and combinations of virulence determinants may all contribute to the observed clinical phenotype.

Overall, the limited availability and heterogeneity of clinical information in the included studies preclude robust conclusions regarding direct associations between specific genetic profiles and disease severity. Nevertheless, the available evidence demonstrates the value of integrating clinical metadata with molecular characterization. The combination of clinical information, *stx* subtyping, virulence profiling, and genomic characterization would allow future studies to investigate more precisely which combinations of bacterial determinants are associated with different clinical outcomes, while avoiding interpretation of individual markers as independent predictors of disease severity.

### Antimicrobial resistance in STEC: Current evidence and limitations

Antimicrobial resistance (AMR) was investigated in only a subset of the studies included in this review, using phenotypic antimicrobial susceptibility testing, detection of resistance determinants, or both. In several studies, AMR information was not reported (NR), not tested (NT), or considered not applicable (NA). Therefore, the available evidence on AMR should be interpreted cautiously and cannot be considered representative of the overall prevalence of antimicrobial resistance among STEC isolates. Nevertheless, the identification of phenotypic resistance, multidrug-resistant profiles, and acquired resistance determinants among STEC from animal, food, and human sources indicates that AMR represents an additional characteristic of epidemiological relevance within this pathotype.

Among studies providing phenotypic resistance results specifically attributable to STEC isolates, resistance to different antimicrobial agents was observed, although the profiles varied according to the populations investigated and the antimicrobial panels tested. Rather than indicating predominance of a particular resistance phenotype across STEC, the recurrence of findings involving tetracyclines, aminoglycosides, sulfonamides/trimethoprim, and other antimicrobial classes should be interpreted in the context of the selective pressures to which enteric bacteria may be exposed in human and animal populations. This consideration is particularly relevant to the animal-origin isolates represented in the present review. Globally, tetracyclines remain the most widely used antimicrobial class in animals, while sulfonamides/trimethoprim and aminoglycosides are also among the antimicrobial classes used in animal health WHO [70].

The overlap between antimicrobial classes used in food-producing animals and resistance phenotypes or determinants identified in animal-associated STEC provides a biologically plausible context for some of the patterns observed in the retrieved literature. Antimicrobial exposure can exert selective pressure on intestinal bacterial populations, favoring the maintenance and expansion of resistant bacteria or resistance determinants. International guidance therefore recognizes antimicrobial use in food-producing animals as an important component of AMR selection and recommends reducing unnecessary use of medically important antimicrobials WHO [69]. However, the studies included in this review generally did not quantify antimicrobial use or previous exposure in the animals or production systems from which STEC isolates were obtained. Consequently, the resistance profiles observed here cannot be causally attributed to veterinary antimicrobial use, and this relationship should be regarded as an epidemiologically plausible interpretation requiring direct investigation.

Multidrug-resistant (MDR) profiles were reported in some investigations. For interpretative consistency in this review, MDR was considered according to the standardized definition proposed by Magiorakos et al. [38], namely acquired non-susceptibility to at least one antimicrobial agent in three or more antimicrobial categories. However, because the included studies differed in the antimicrobial panels tested and did not always provide sufficient information to independently apply this criterion, isolates were not retrospectively reclassified. Therefore, the term MDR was retained only when explicitly reported by the original study and was interpreted according to the criteria adopted by its authors. Lopes et al. [34] provides an illustrative example of the importance of defining the analytical denominator. Among 213 *E. coli* isolates obtained from conventional broilers, 35 were identified as STEC, while 21 *E. coli* isolates were classified as MDR. One of these 21 MDR isolates belonged to the STEC pathotype. Because the STEC isolates underwent the reported antimicrobial susceptibility testing, the corresponding STEC-specific MDR proportion was 1/35 (2.86%), whereas the original value of 1/21 (4.76%) represents the proportion of STEC among the MDR *E. coli* isolates and should not be interpreted as the prevalence of MDR among STEC. From an epidemiological perspective, the occurrence of MDR among enteric bacteria from food-producing animals is relevant beyond the treatment of STEC infection itself, because these bacteria may participate in broader reservoirs of resistance determinants within animal-associated microbial communities. Accordingly, AMR in STEC should be considered within the wider ecology of antimicrobial resistance at the animal–food–human interface rather than exclusively as a characteristic affecting clinical management of STEC disease. This interpretation is consistent with integrated One Health surveillance approaches for antimicrobial resistance in foodborne bacteria.

In addition to phenotypic resistance, some studies identified antimicrobial resistance determinants through molecular or genomic analyses. Lopes et al. [35], for example, detected *mcr-1*, a determinant associated with colistin resistance, in 2/35 STEC isolates (5.7%). The occurrence of this determinant is epidemiologically relevant because *mcr-1* may be associated with transferable plasmids and therefore contribute to horizontal dissemination of colistin resistance. However, the data extracted from Lopes et al. [35] do not establish that *mcr-1* was plasmid-borne in the STEC isolates identified in that study. More broadly, resistance genes and mobile genetic elements were not systematically investigated across the included studies, limiting conclusions regarding their contribution to AMR dissemination in STEC. Nevertheless, the detection of acquired resistance determinants in STEC emphasizes that virulence and antimicrobial resistance can coexist within the same bacterial population. From a surveillance perspective, genomic approaches are particularly valuable in this context because they can simultaneously characterize virulence determinants, resistance genes, and their potential association with mobile genetic elements.

The studies in which both genotypic and phenotypic AMR information was available also demonstrate that detection of a resistance determinant does not necessarily predict phenotypic resistance. Bai et al. [7], for example, detected *mdf(A)* in three STEC isolates that remained phenotypically susceptible to the antimicrobials tested, whereas Mussio et al. [49] reported both concordant and discordant genotype–phenotype findings depending on the resistance determinant and antimicrobial evaluated. These observations reinforce the complementary nature of phenotypic and genomic approaches: genomic detection can reveal the resistance gene repertoire, whereas phenotypic testing establishes whether resistance is expressed under the conditions evaluated. Integrating both approaches therefore provides a more informative assessment than either method alone.

STEC-specific antimicrobial resistance findings were reported in studies involving isolates from animal, food, and human sources. However, no formal comparison of resistance frequencies among source categories was performed in this review. Differences in the number of studies, antimicrobial panels, sampling strategies, analytical populations, and susceptibility-testing methods precluded reliable source-specific comparisons. Therefore, the available evidence does not support the conclusion that STEC isolates from one source had higher resistance frequencies than those from another. At the same time, the presence of resistance determinants across different components of the animal–food–human interface reinforces the value of coordinated and integrated AMR surveillance within a One Health framework WHO [67, 68]. Resistant enteric bacteria and their genetic determinants may circulate across ecological compartments associated with humans, animals, and the food chain, although the direction and magnitude of these processes cannot be established from the present dataset.

Overall, the available evidence indicates that antimicrobial resistance, including MDR profiles and resistance-associated genetic determinants, has been documented among STEC isolates in different contexts. The main contribution of these findings is therefore not an estimate of how common AMR is among STEC, but the demonstration that resistance determinants and virulence characteristics may coexist in strains circulating at animal, food, and human interfaces. The occurrence of resistance to antimicrobial classes also used in animal production provides a relevant context for investigating the role of antimicrobial selective pressure, particularly in food-producing animals; however, establishing such relationships will require studies that integrate isolate-level AMR characterization with quantitative information on antimicrobial exposure and production practices. Future studies using standardized antimicrobial susceptibility testing and reporting approaches, with results clearly stratified according to pathotype, source, and antimicrobial exposure, are needed to improve comparability and strengthen surveillance of AMR in STEC across human, animal, food, and environmental interfaces.

### One health interface in STEC dissemination: Integration between reservoirs, environment, and the food chain

The distribution of STEC across the included studies clearly highlights the multifactorial and interconnected nature of its dissemination, reinforcing the importance of the One Health approach in understanding this pathogen. The data from this review show that STEC was identified across animal, food, environmental, and human sources, highlighting the interconnected interfaces relevant to its epidemiology. The epidemiological significance of this pattern lies not simply in the occurrence of STEC in different compartments, but in the possibility that information generated in each of them contributes complementary evidence for understanding pathogen circulation, contamination events, and risks along the food chain.

Rather than considering these sources independently, the findings across the included studies indicate the relevance of their interfaces within STEC epidemiology. Animal-associated studies documented STEC in food-producing species, while food and environmental investigations demonstrated its occurrence in products and production-related settings, and clinical studies characterized strains recovered from humans [1, 12, 17, 24]. Together, these findings provide complementary evidence across different components of the animal–food–environment–human interface, although the present review was not designed to establish direct transmission pathways among these compartments. This distinction is important because detection of related virulence profiles, serotypes, or sequence types in different sources may generate epidemiological hypotheses, but does not by itself demonstrate transmission between them.

Genomic characterization provides an additional means of investigating these interfaces by enabling comparison of isolates from different sources. Studies including isolates from animals, food, and humans have demonstrated genetic relationships among strains from distinct epidemiological contexts, illustrating the value of integrating genomic and epidemiological information in STEC surveillance [26]. Nevertheless, genetic relatedness alone does not establish the direction or route of transmission, and such relationships should be interpreted together with epidemiological and source information. In this context, higher-resolution genomic approaches can complement conventional surveillance by helping to identify clusters, distinguish closely related strains, and investigate potential epidemiological connections when appropriate metadata are available.

Additionally, the detection of antimicrobial-resistant and multidrug-resistant STEC in some of the investigated matrices adds another dimension to the One Health relevance of this pathogen. However, because antimicrobial resistance was not uniformly assessed across the included studies, its contribution to STEC dissemination across these interfaces cannot be determined from the present dataset. The coexistence of virulence and antimicrobial-resistance determinants nevertheless illustrates why STEC surveillance may benefit from integration with broader AMR monitoring at the animal–food–human interface. Such integration is consistent with international strategies that recognize antimicrobial resistance as a multisectoral problem requiring coordinated surveillance across human and animal health and food-production systems WHO [67–69].

Overall, the findings of this review support One Health as an integrative framework for STEC surveillance rather than as evidence of a single continuous transmission pathway. The principal contribution of this perspective is therefore the integration of otherwise fragmented information from animal, food, environmental, and human studies. Combining information from animal, food, environmental, and human sources with molecular and genomic characterization may improve the identification of epidemiological connections and support coordinated monitoring, prevention, and control strategies across food production and public health systems.

### Limitations and future implications

This review presents several limitations that should be considered when interpreting the results. The main limitation relates to the methodological heterogeneity among the included studies, particularly regarding the techniques used for detection and characterization of STEC strains. While some studies employed advanced molecular methods, such as whole-genome sequencing (WGS) and toxin subtyping analyses [6, 33, 43], others relied predominantly on conventional techniques, including targeted PCR and phenotypic assays [2, 13]. This variation limits direct comparability among studies because the methods differed in their analytical targets, resolution, and characterization capacity. Furthermore, not all studies investigated the same virulence determinants, stx subtypes, antimicrobial-resistance profiles, or mobile genetic elements. Consequently, absence of reporting could not be interpreted as absence of a particular characteristic, limiting comparisons based on common analytical denominators.

Another important limitation concerns the absence or inconsistency of clinical data reporting, observed in the majority of the included studies. Although some investigations reported both virulence profiles and clinical manifestations [5, 20], most studies focused primarily on the microbiological characterization of isolates without providing detailed clinical descriptions. This gap limits a deeper understanding of the relationship between genotype and clinical phenotype, particularly regarding progression to severe outcomes such as hemolytic uremic syndrome (HUS). Future studies combining standardized clinical metadata with detailed molecular characterization may therefore improve assessment of associations between specific virulence profiles and disease severity.

In addition, a geographic concentration of studies was observed, with a predominance of research conducted in Brazil and other countries in the Americas, which may influence the global representativeness of the data. Although studies from different regions were included [7, 26], the uneven distribution of evidence limits broader inferences about the global epidemiology of STEC. Furthermore, the restriction to studies published in English and the predefined search strategy may have resulted in the omission of relevant evidence, particularly from underrepresented geographic regions. The search strategy also did not systematically incorporate additional terminology such as VTEC, EHEC, shigatoxigenic, specific non-O157 serogroups, or additional source-related terms. Accordingly, the findings should be interpreted as a descriptive synthesis of the literature retrieved through the predefined strategy rather than as an exhaustive representation of STEC research published during the period evaluated.

Another relevant point refers to the variability in genetic characterization of strains, particularly regarding the identification of *stx* subtypes and other virulence factors. Recent studies have demonstrated an increasing diversity of Shiga toxin subtypes, including rare and emerging variants [27, 33, 52], reinforcing the need for methodological standardization in subtyping and nomenclature, as proposed by Scheutz et al. [54]. The lack of such standardization may hinder comparisons between studies and compromise the consolidation of knowledge in this field. In addition, differences between targeted molecular assays and higher-resolution genomic approaches influence the level of genetic information that can be obtained, reinforcing the importance of clearly reporting analytical targets and methods when comparing STEC populations across studies.

Additionally, because of the substantial heterogeneity in study designs and objectives, no formal risk-of-bias scoring system was applied. Methodological limitations were instead considered descriptively, and the findings should therefore be interpreted as a descriptive synthesis of the available evidence rather than as a formally weighted assessment of study quality.

Antimicrobial resistance represents an additional limitation of the available evidence. AMR was not uniformly investigated across the included studies, and differences in antimicrobial panels, phenotypic methods, genomic approaches, analytical populations, and reporting strategies limited comparisons among studies and sources. Importantly, studies involving animal-origin STEC generally did not provide quantitative information on antimicrobial exposure or antimicrobial-use practices in the production systems from which the isolates originated. Therefore, although the resistance findings can be discussed in the broader context of selective pressures associated with antimicrobial use in animal production WHO [68, 69], the present review cannot establish causal relationships between veterinary antimicrobial use and the resistance profiles observed. Future studies integrating phenotypic susceptibility, resistance-genome characterization, source information, and antimicrobial-use data would provide stronger evidence for investigating these relationships WHO [68–70].

From the perspective of future implications, the findings of this review support further integration of molecular and epidemiological approaches. Where appropriate to the study objectives and available resources, high-resolution approaches such as WGS may provide complementary information on genetic diversity, virulence determinants, and genomic relationships among STEC strains [26, 61]. Additionally, the identification of antimicrobial resistance genes and mobile genetic elements among some of the investigated strains supports the value of further investigation and surveillance of these determinants across different matrices, while recognizing that AMR was assessed in only a limited and heterogeneous subset of the included studies. Combining genomic information with phenotypic, epidemiological, clinical, and antimicrobial-exposure data may provide a more informative framework for interpreting the public-health significance of individual STEC strains than any of these data sources considered independently.

The detection of STEC across animal, food, environmental, and human sources also supports the value of studies integrating these interfaces within a One Health framework. However, because the included studies differed substantially in design and were not generally structured to evaluate transmission across all of these compartments simultaneously, the present findings do not establish direct transmission pathways among them. Future investigations integrating epidemiological, molecular, and source-related information may help clarify these relationships and inform surveillance and control strategies [17, 40].

Finally, future studies would benefit from more detailed and standardized clinical metadata combined with molecular and genomic characterization, allowing more robust investigation of potential associations between genetic profiles and clinical manifestations. Greater standardization of STEC characterization and reporting across human, animal, food, and environmental studies would also improve comparability among investigations and facilitate integration of evidence across these interfaces. Such integration may improve the evidence available to inform the prevention, diagnosis, and control of STEC infections.

## Conclusion

This descriptive review identified studies investigating STEC across diverse human, animal, food, and environmental contexts, with substantial genetic and epidemiological heterogeneity among the included studies. The available evidence was geographically uneven, with a marked concentration of studies from the Americas, particularly Brazil, and therefore should not be interpreted as a comprehensive representation of global STEC epidemiology.

The included studies reported substantial diversity in Shiga toxin subtypes, particularly among *stx2* variants, together with diverse serotypes, sequence types, and additional virulence-associated determinants. Antimicrobial resistance data were available for only a subset of the included studies and showed heterogeneous phenotypic and genotypic profiles when reported.

Overall, the findings reinforce the importance of integrated STEC surveillance across human, animal, food, and environmental interfaces. Given the descriptive nature and heterogeneity of the available evidence, these findings should not be interpreted as demonstrating quantitative associations between specific sources, virulence profiles, and clinical outcomes. Further studies from underrepresented geographic regions, using standardized epidemiological and molecular approaches, are needed to improve the comparability of data and provide a more representative understanding of STEC distribution and diversity.

## Data Availability

No datasets were generated or analysed during the current study.
